# Supplementary material for: Decasubstituted Pillar[5]arene Derivatives Containing L-Tryptophan and L-Phenylalanine Residues: Non-Covalent Binding and Release of Fluorescein from Nanoparticles
Source: Int J Mol Sci. 2023 Apr 22;24(9):7700. doi: 10.3390/ijms24097700 (PMC10178471; doi:10.3390/ijms24097700)
Supplement: Supplementary file 1 [file ijms-24-07700-s001.zip › ijms-2345690-supplementary.pdf]

Supplementary Materials

Decasubstituted Pillar[5]arene Derivatives Containing *L*–Tryptophan and *L*–Phenylalanine Residues: Non-Covalent Binding and Release of Fluorescein from Nanoparticles

Vildan Sultanaev [1], Luidmila Yakimova [1,*], Anastasia Nazarova [1], Olga Mostovaya [1], Igor Sedov [1], Damir Davletshin [2], Elvina Gilyazova [2], Emil Bulatov [2], Zhang-Ting Li [3], Dan-Wei Zhang [3] and Ivan Stoikov [1,4,*]

1. A.M. Butlerov Chemistry Institute, Kazan Federal University, 18 Kremlyovskaya Str., 420008 Kazan, Russia; vildan_sultanaev@mail.ru (V.S.)
2. Institute of Fundamental Medicine and Biology, Kazan Federal University, 18 Kremlyovskaya Str., 420008 Kazan, Russia
3. Department of Chemistry, Shanghai Key Laboratory of Molecular Catalysis and Innovative Materials, Fudan University, 2205 Songhu Road, Shanghai 200438, China
4. Federal State Budgetary Scientific Institution «Federal Center for Toxicological, Radiation, and Biological Safety», Nauchny Gorodok, 2, Kazan 420075, Russia

* Correspondence: mila.yakimova@mail.ru (L.Y.); ivan.stoikov@mail.ru (I.S.); Tel.: +7-843-233-7241 (L.Y. & I.S.)

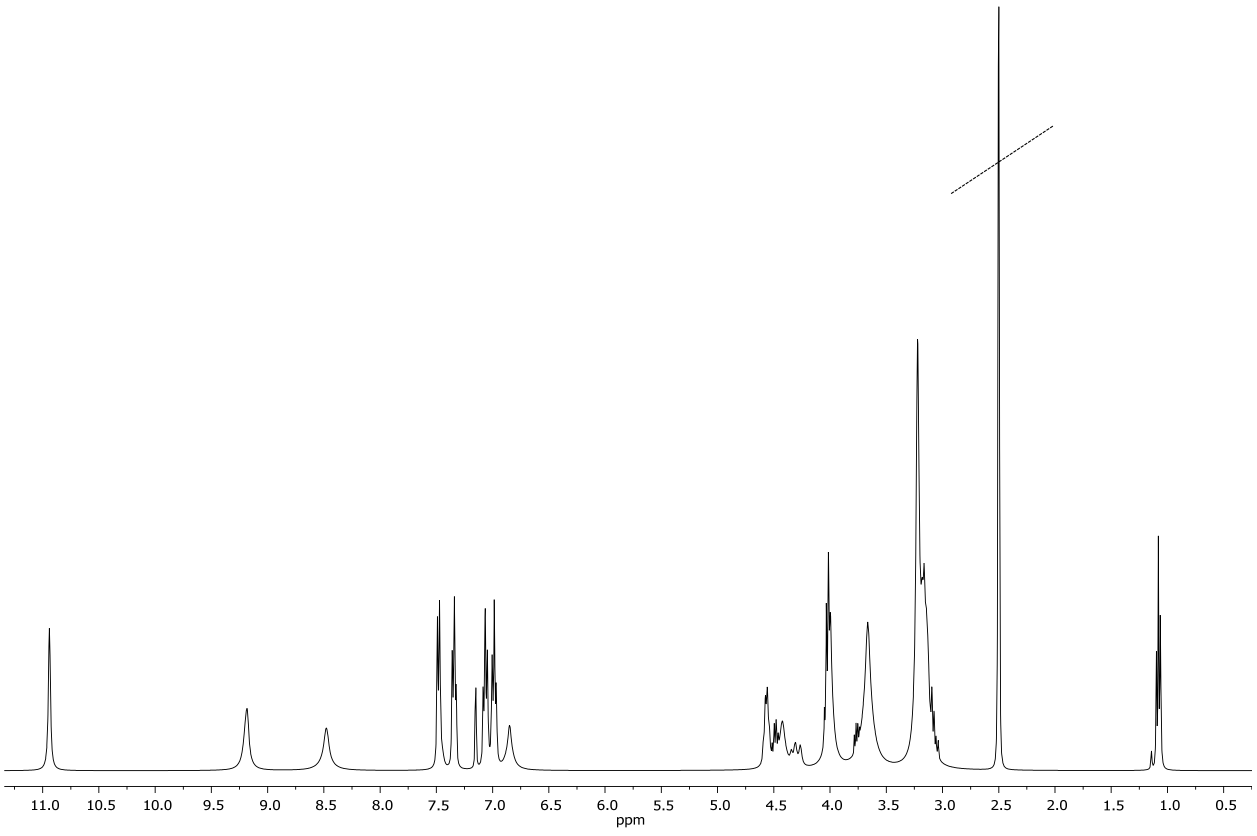

Figure S1. ^{1}H NMR spectrum of 4,8,14,18,23,26,28,31,32,35-Deca-[(*N*-[2-dimethyl({ethoxycarbonyl[*S*-indole-3-yl-methyl]methyl}aminocarbonylmethyl)ammonio]ethyl)aminocarbonylmethoxy]-pillar[5]arene decabromide (**2**), DMSO-d_6, 298 K, 400 MHz.

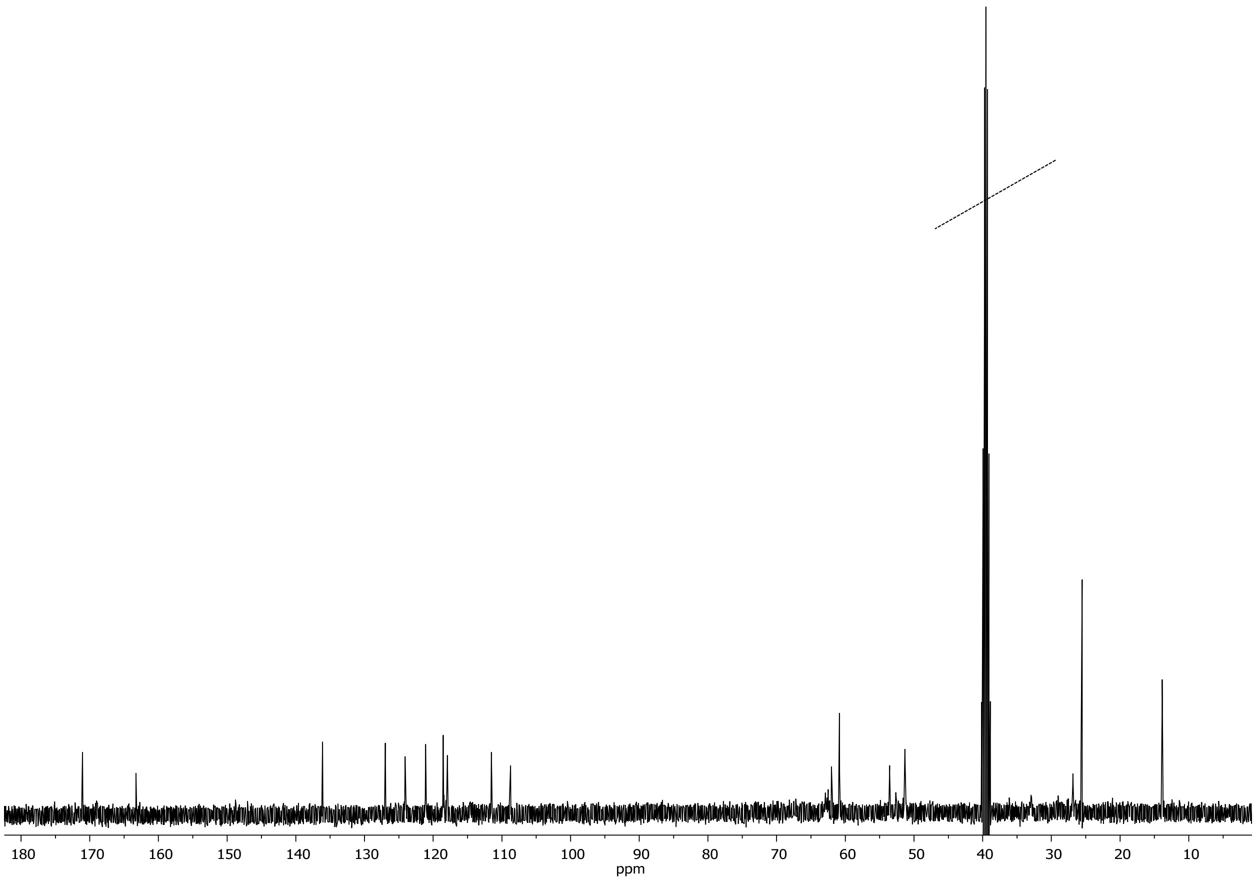

Figure S2. ^{13}C NMR spectrum of 4,8,14,18,23,26,28,31,32,35-Deca-[(*N*-[2-dimethyl({ethoxycarbonyl [*S*-indole-3-yl-methyl]methyl}aminocarbonylmethyl)ammonio]ethyl)aminocarbonylmethoxy]-pillar[5]arene decabromide (**2**), DMSO-d_6, 298 K, 100 MHz.

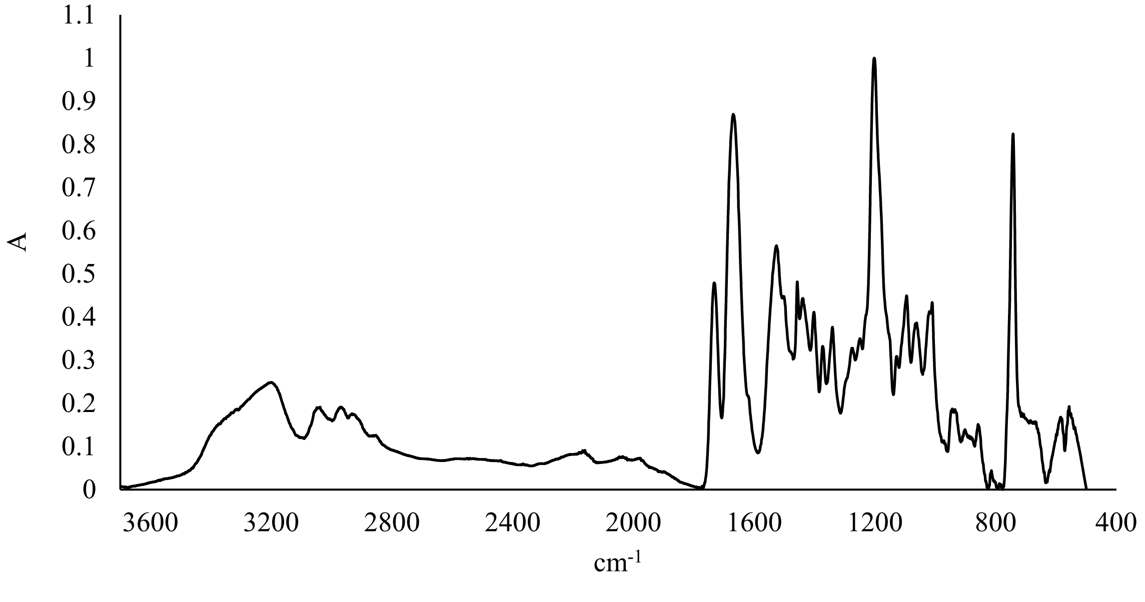

Figure S3. IR spectrum of 4,8,14,18,23,26,28,31,32,35-Deca-[(*N*-[2-dimethyl({ethoxycarbonyl [*S*-indole-3-yl-methyl]methyl}aminocarbonylmethyl)ammonio]ethyl)aminocarbonylmethoxy]-pillar[5]arene decabromide (**2**).

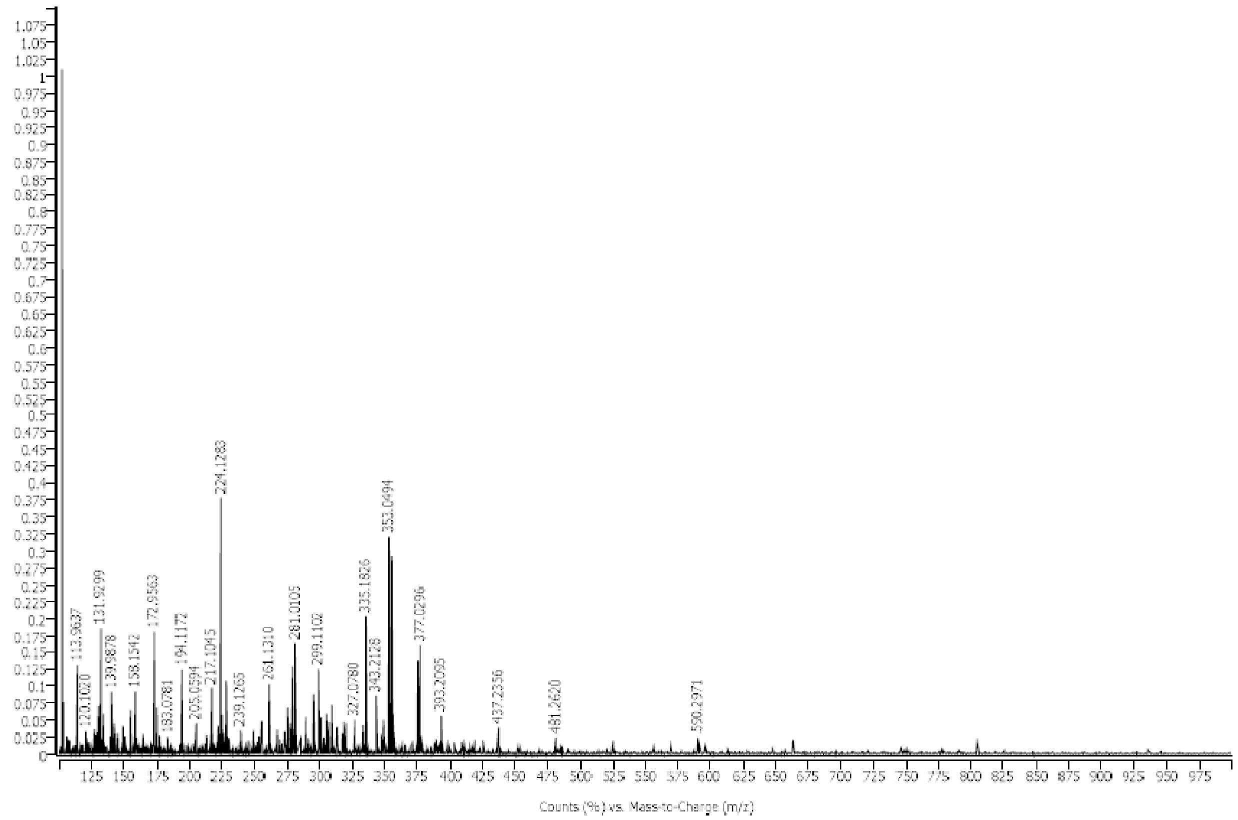

Figure S4. Mass spectrum (ESI HRMS) of 4,8,14,18,23,26,28,31,32,35-Deca-[(*N*-[2-dimethyl({ethoxycarbonyl[*S*-indole-3-yl-methyl]methyl}aminocarbonylmethyl)ammonio]ethyl) aminocarbonylmethoxy]-pillar[5]arene decabromide (**2**).

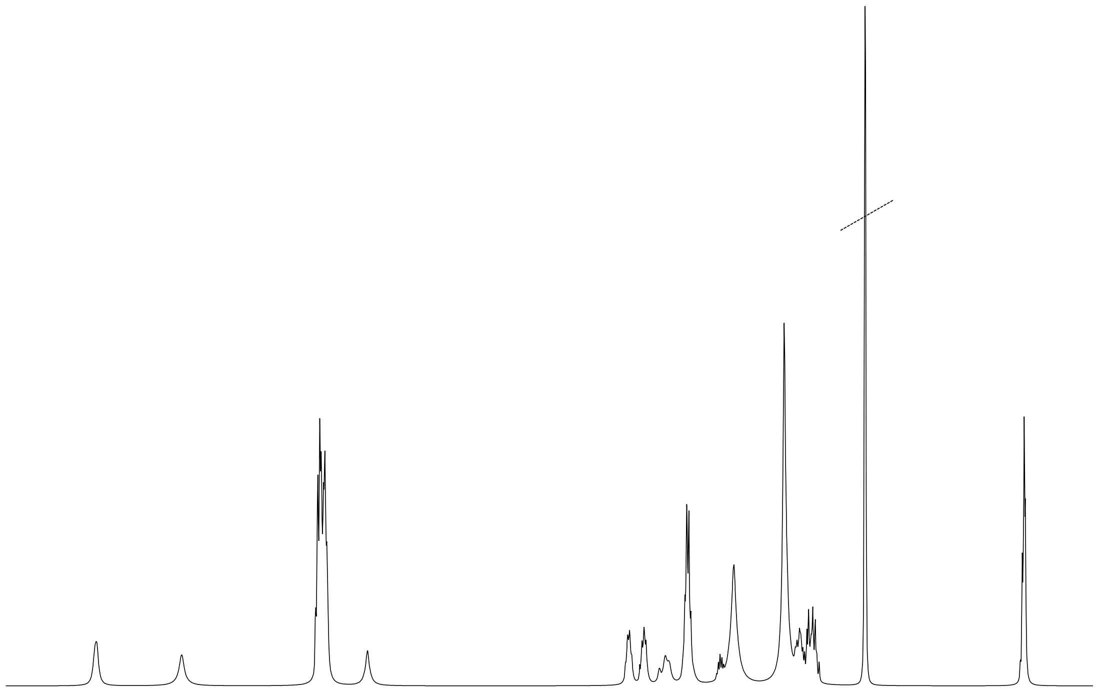

Figure S5. ^{1}H NMR spectrum of 4,8,14,18,23,26,28,31,32,35-Deca-[(*N*-[2-dimethyl({ethoxycarbonyl[*S*-benzyl]methyl}aminocarbonylmethyl)ammonio]ethyl)amino carbonylmethoxy]-pillar[5]arene decabromide (**3**), DMSO-d_6, 298 K, 400 MHz.

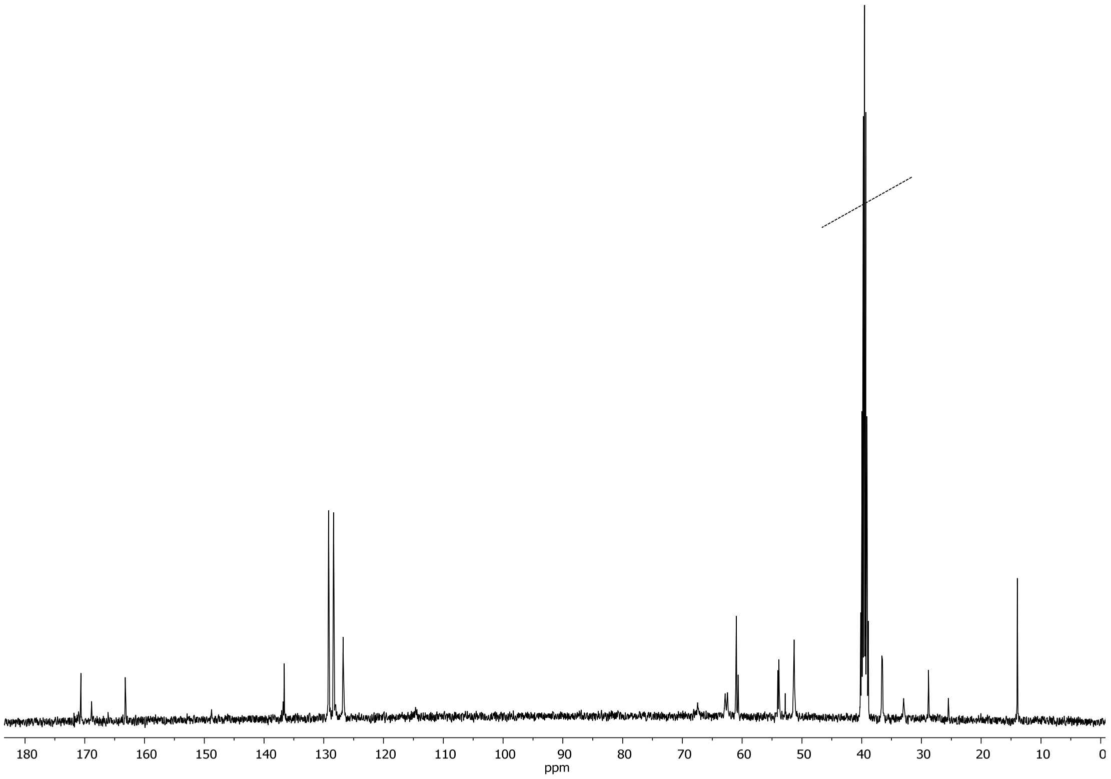

Figure S6. ^{13}C NMR spectrum of 4,8,14,18,23,26,28,31,32,35-Deca-[(*N*-[2-dimethyl({ethoxycarbonyl [*S*-benzyl]methyl}aminocarbonylmethyl)ammonio]ethyl)aminocarbonylmethoxy]-pillar[5]arene decabromide (**3**), DMSO-d_6, 298 K, 100 MHz.

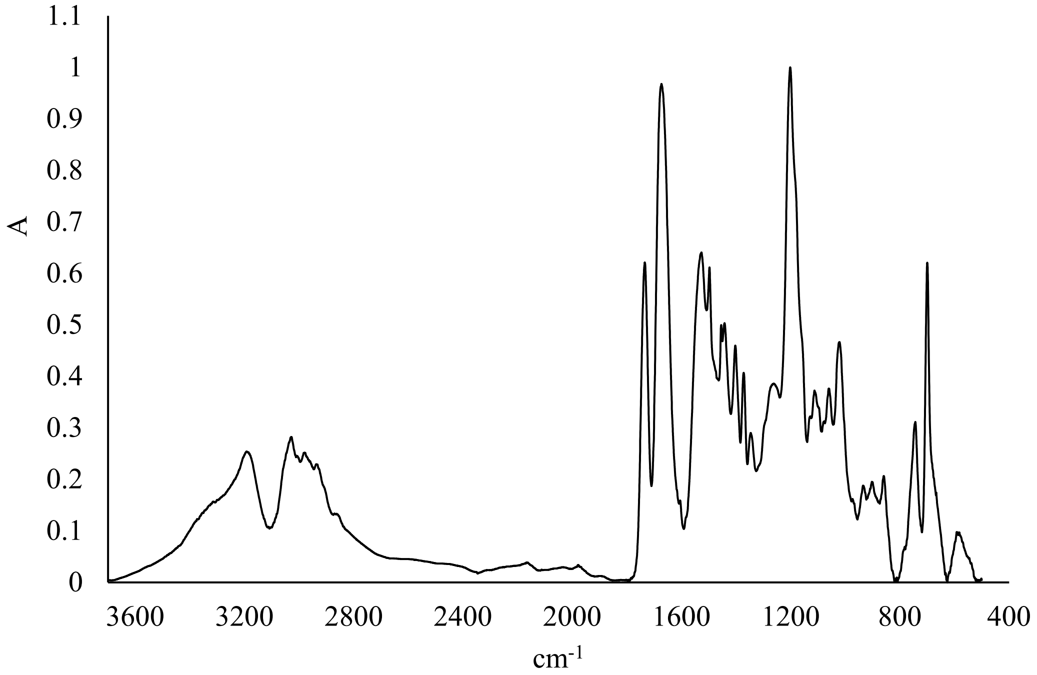

Figure S7. IR spectrum of 4,8,14,18,23,26,28,31,32,35-Deca-[(*N*-[2-dimethyl({ethoxycarbonyl[*S*-benzyl] methyl}aminocarbonylmethyl)ammonio]ethyl)aminocarbonylmethoxy]-pillar[5]arene decabromide (**3**).

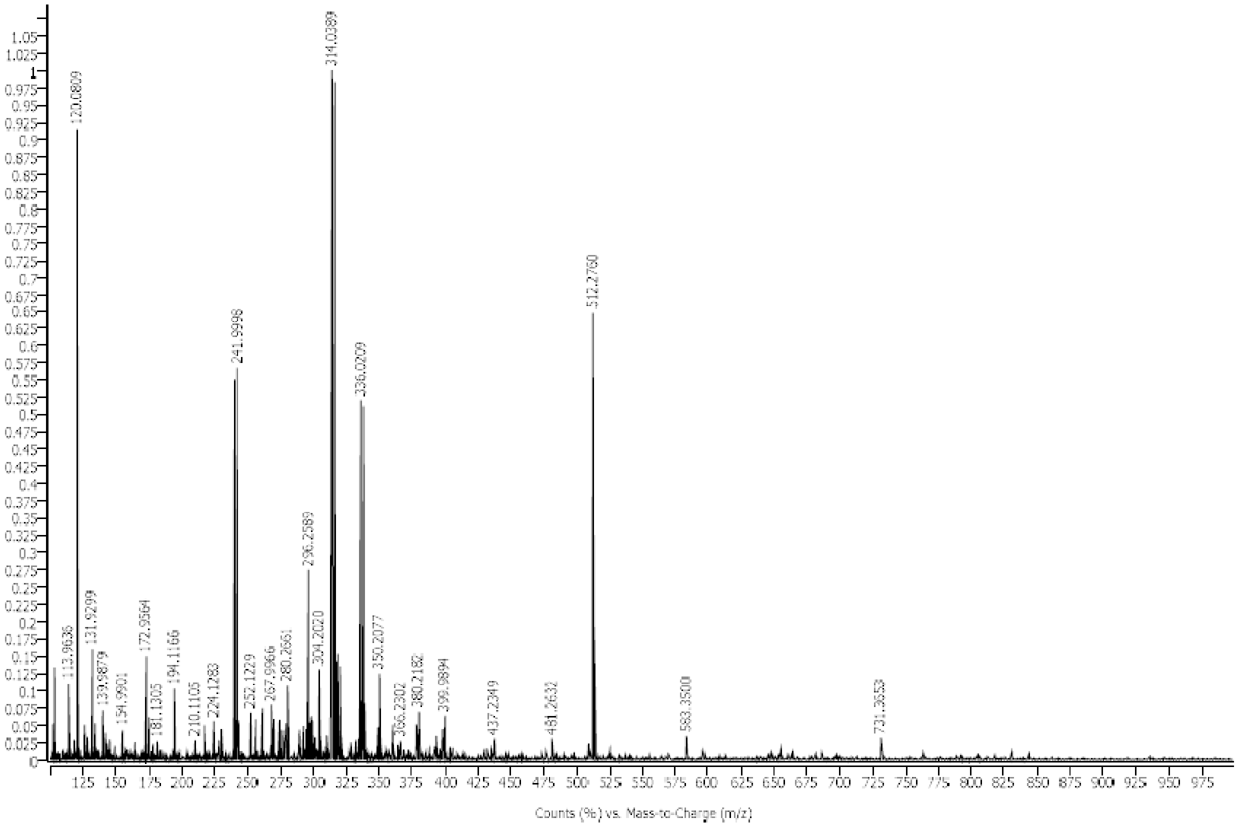

Figure S8. Mass spectrum (ESI HRMS) of 4,8,14,18,23,26,28,31,32,35-Deca-[(*N*-[2-dimethyl ({ethoxycarbonyl[*S*-benzyl]methyl}aminocarbonylmethyl)ammonio]ethyl)aminocarbonylmethoxy]-pillar[5]arene decabromide (**3**).

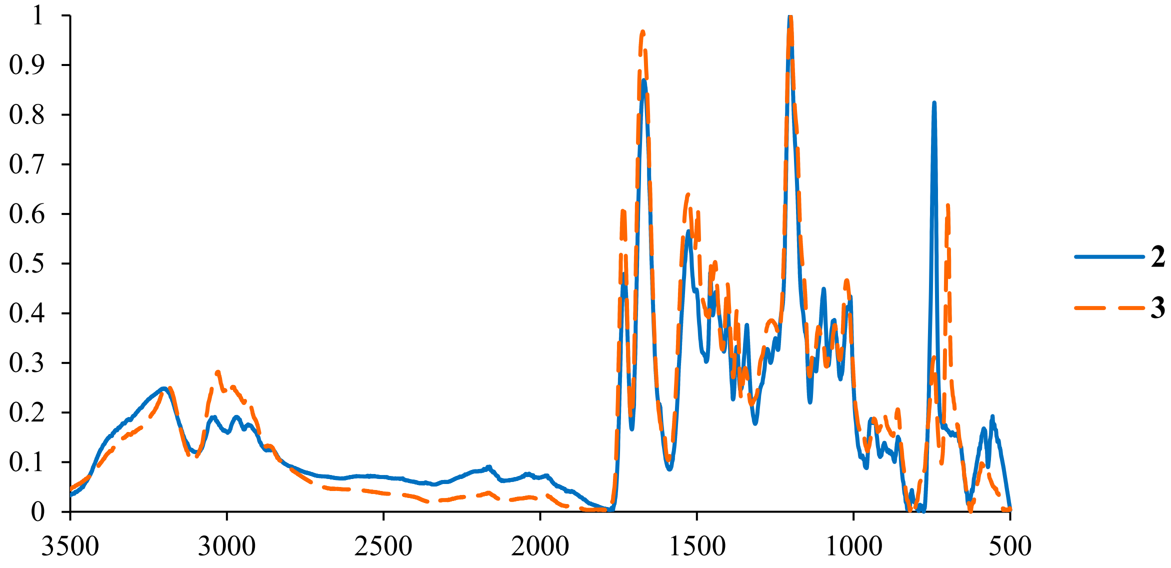

Figure S9. IR spectra of compounds **2** (solid line) and **3** (dashed line).

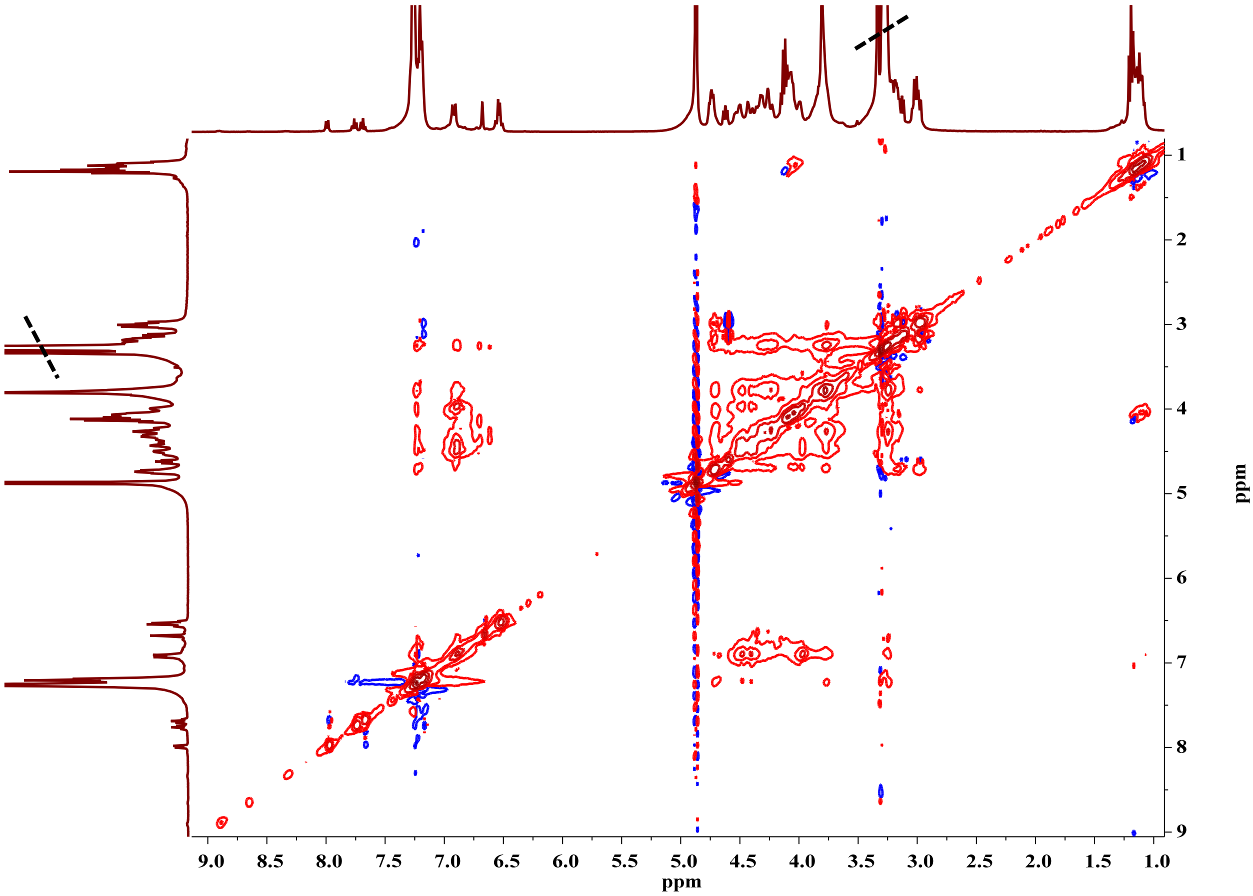

Figure S10. ${}^{1}H$–${}^{1}H$ NOESY NMR spectrum of mixture **3/Fluo** in 1:1 molar ratio (CD_3OD–d_4, 298 K, 400 MHz).

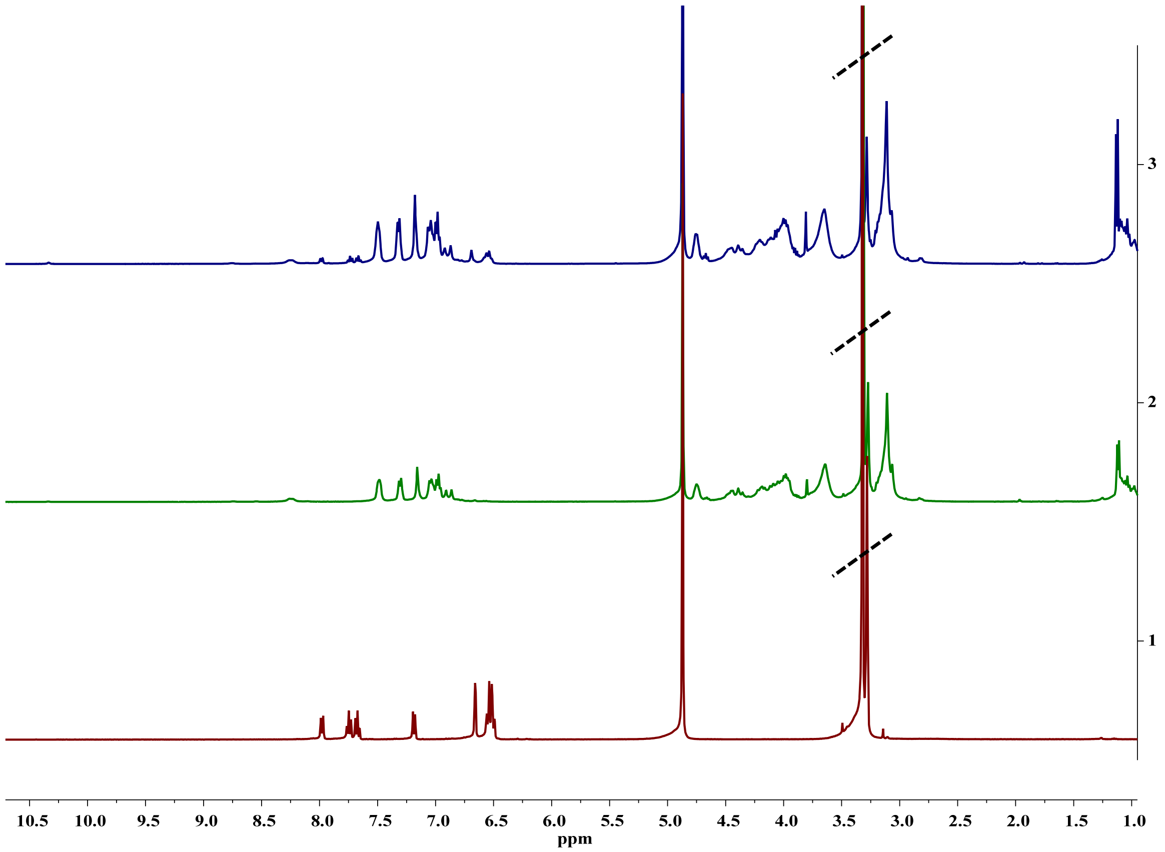

Figure S11. ^{1}H NMR spectra of (1) **Fluo**, (2) **2** and (3) **2/Fluo** in 1:1 molar ratio (CD_3OD-d_4, 298 K, 400 MHz).

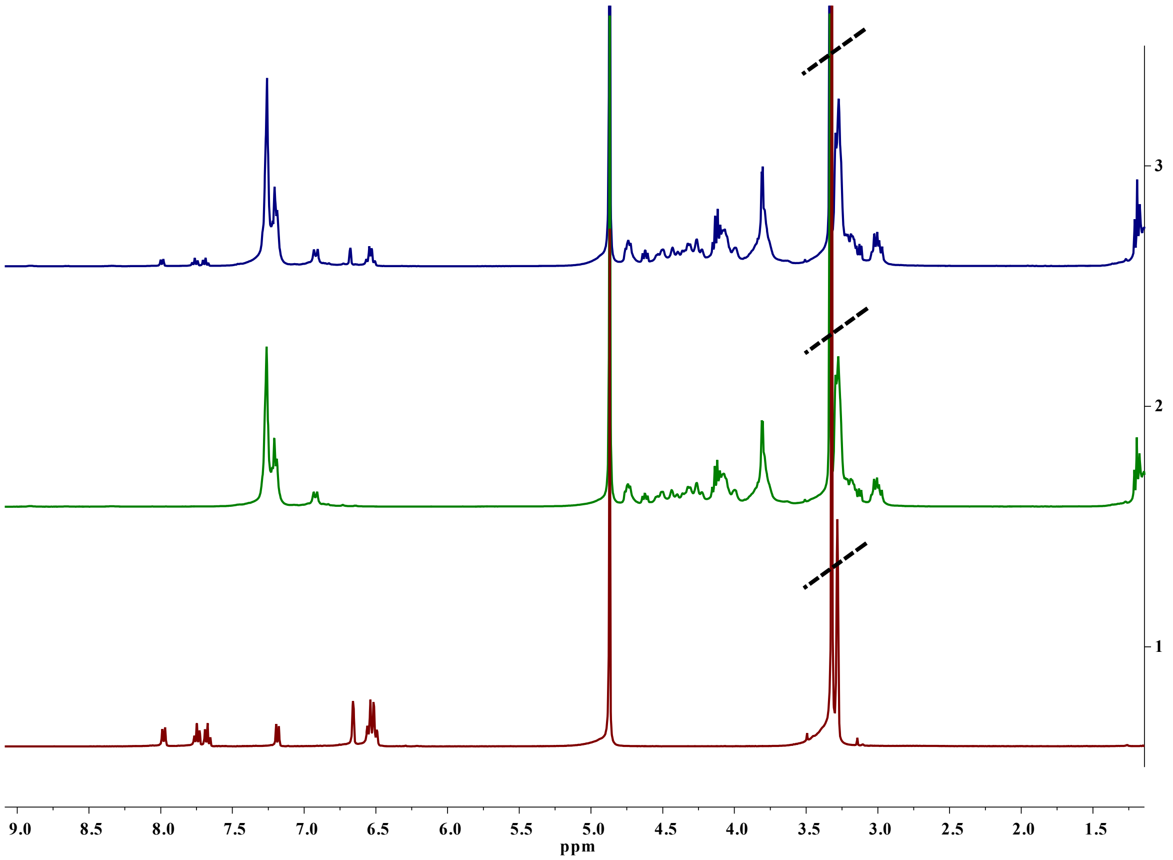

Figure S12. ^{1}H NMR spectra of (1) **Fluo**, (2) **3** and (3) **3/Fluo** in 1:1 molar ratio (CD_3OD-d_4, 298 K, 400 MHz).

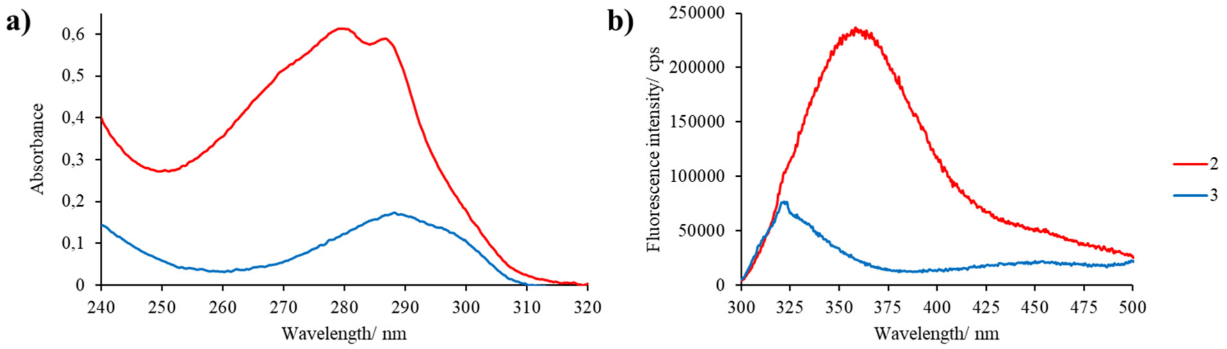

Figure S13. (a) Electronic absorption spectra of macrocycle **2** and **3** (C_2=C_3=1×10^{-5} M), (b) fluorescence spectra of **2** and **3** (C_2=C_3=1×10^{-5} M) in ethanol–water solution.

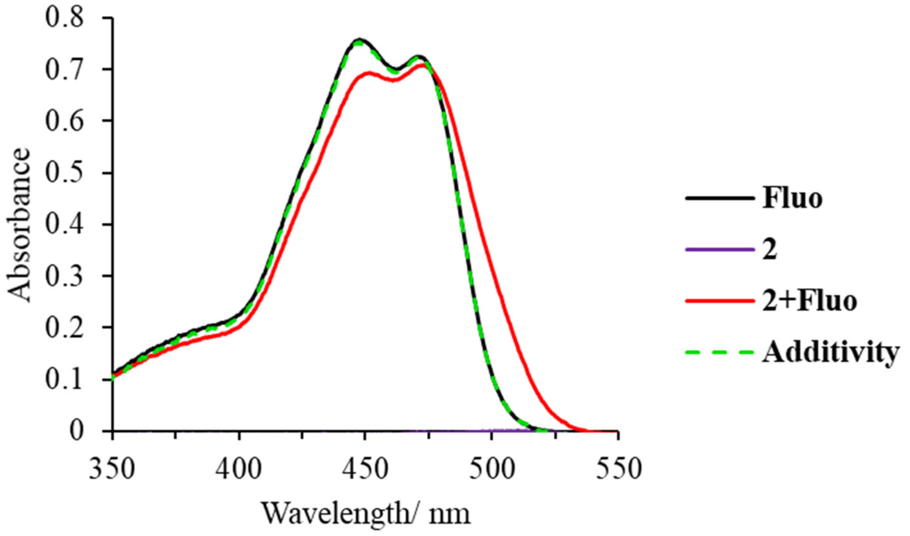

Figure S14. Electronic absorption spectra of macrocycle **2** (C_2=1×10^{-5} M), **Fluo** (C_{Fluo}=1×10^{-5} M) and their mixture in 1:1 molar ratio in ethanol-water solution.

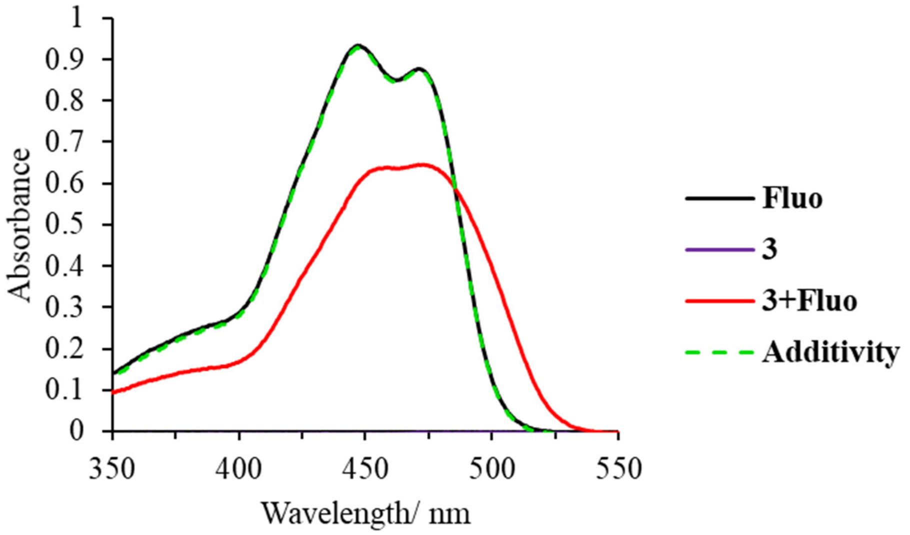

Figure S15. Electronic absorption spectra of macrocycle **3** (C_3=1×10^{-5} M), **Fluo** (C_{Fluo}=1×10^{-5} M) and their mixture in 1:1 molar ratio in ethanol–water solution.

a)

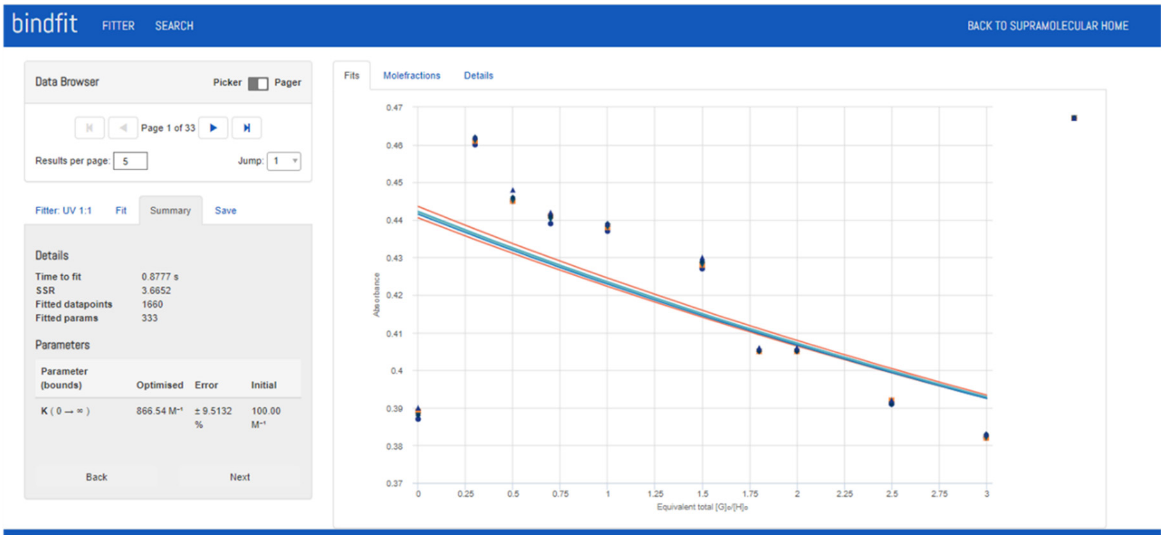

b)

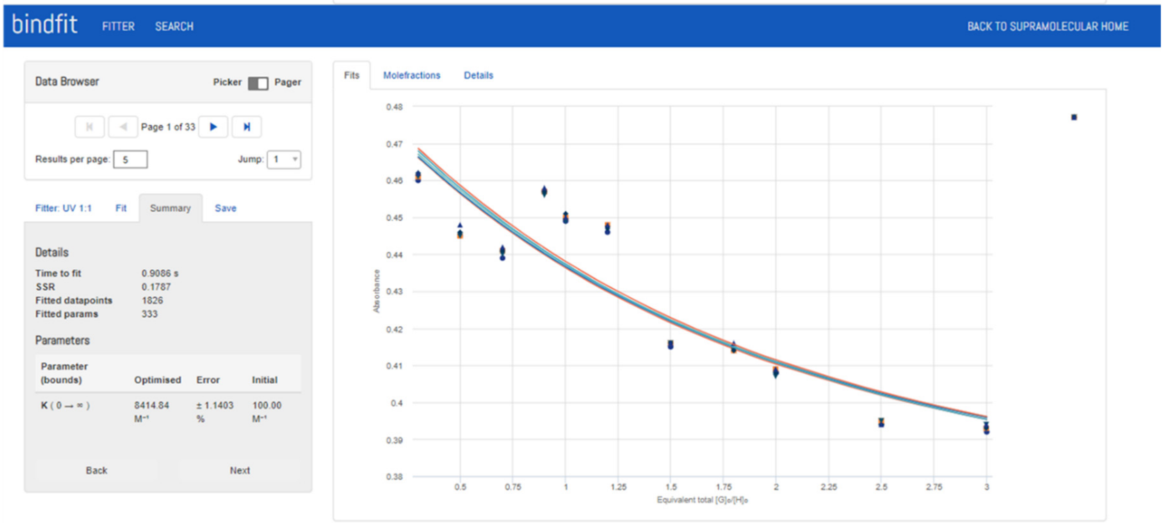

Figure S16. Bindfit (Fit data to 1:1 Host-Guest equilibria) Screenshots taken from the summary window of the website supramolecular.org. This screenshots shows the raw data for UV–vis titration of (a) **2/Fluo** and (b) **3/Fluo** in ethanol–water solution, the data fitted to 1:1 binding model.

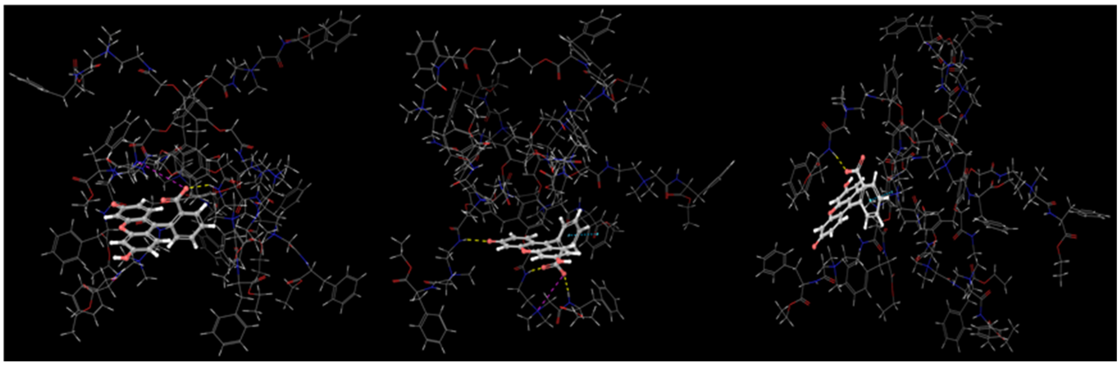

Figure S17. Top 3 scoring poses of fluorescein docked to the different conformations of **3**. Dashed lines represent hydrogen bonding (yellow), electrostatic contacts between charged atoms (purple), π–stacking (blue).

Table S1. pH–data of the individual compounds and the mixtures **2/Fluo** and **3/Fluo** in 1:1 molar ratio in ethanol–water solution (C=1×10^{-4} M).

System	pH
**Fluo**	4.55
**2**	5.75
**2/Fluo**	4.42
**3**	6.62
**3/Fluo**	5.88
EtOH+H_2O	5.05

Table S2. The aggregate sizes (hydrodynamic particle diameters d, nm), intensity distribution of particles formed as a result of self–assembly of compounds **2** and **3** in ethanol-water and water solvents respectively, polydispersity index (PDI) at different concentrations.

C, mol/L	**2**		**3**	
	PDI	d, nm	PDI	d, nm
1 × 10^{-4}	0.36±0.04	117±19	0.34±0.04	223±22
1 × 10^{-5}	N/A*	N/A*	0.40±0.02	247±17
1 × 10^{-6}	N/A*	N/A*	0.43±0.12	364±159

* N/A – not applicable

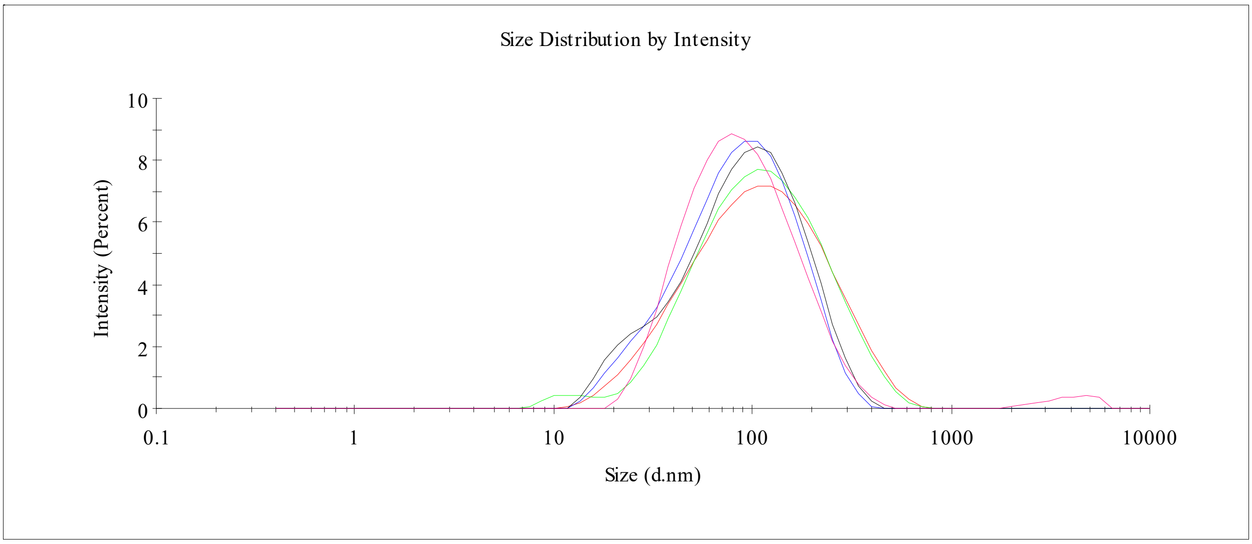

Figure S18. Size distribution of self-associates of macrocycle **2** in ethanol–water solvent system (C_2=1 × 10^{-4}).

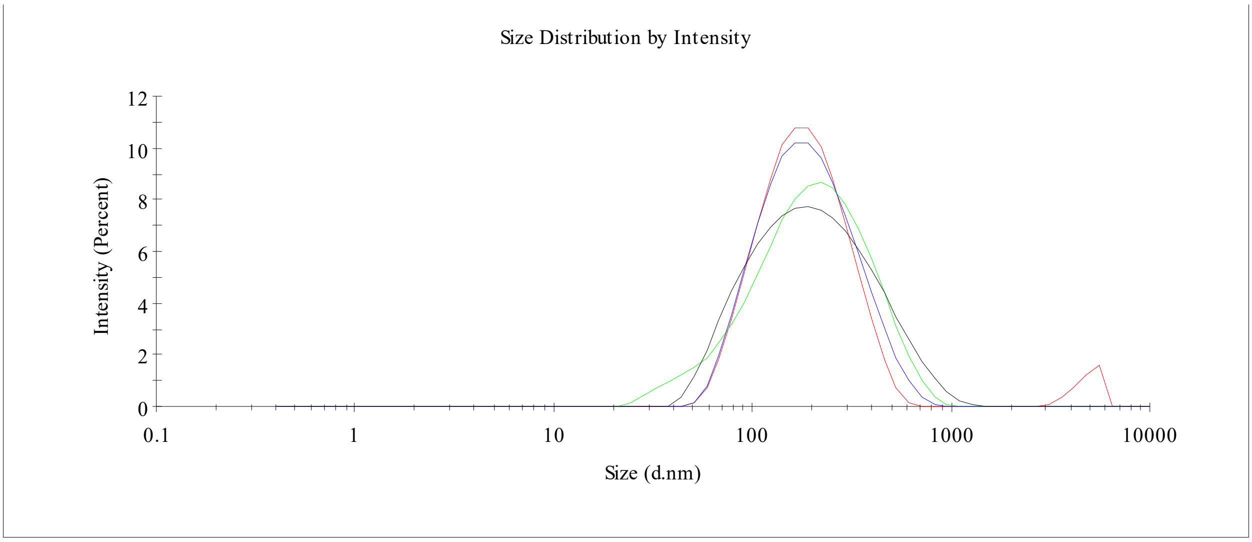

Figure S19. Size distribution of self-associates of macrocycle **3** in water (C_3=1 × 10^{-4} M).

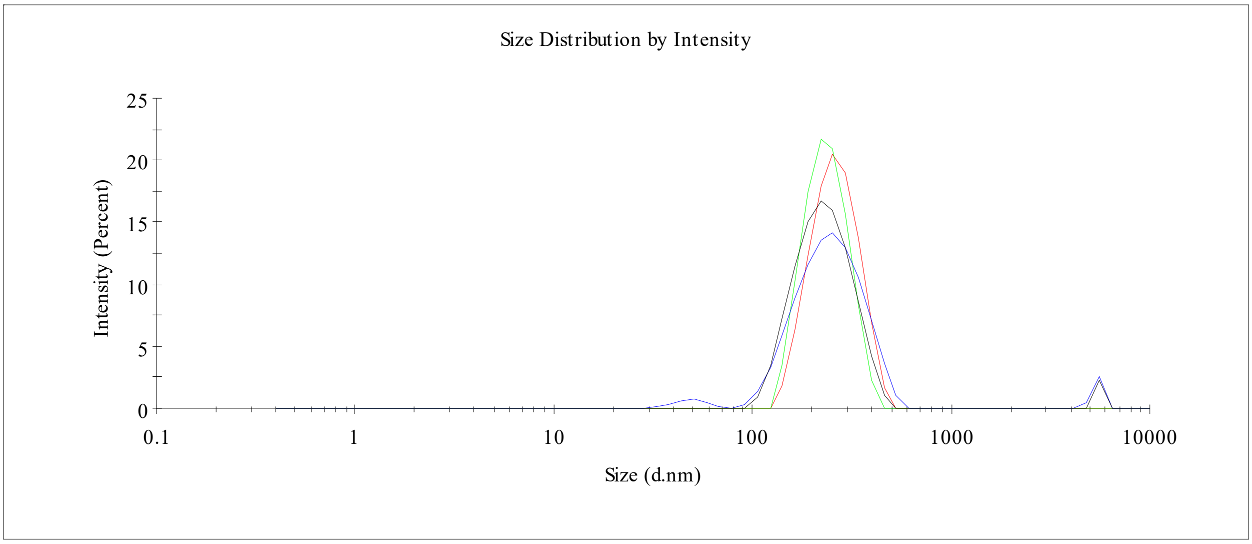

Figure S20. Size distribution of self-associates of macrocycle **3** in water (C_3=1 × 10^{-5} M).

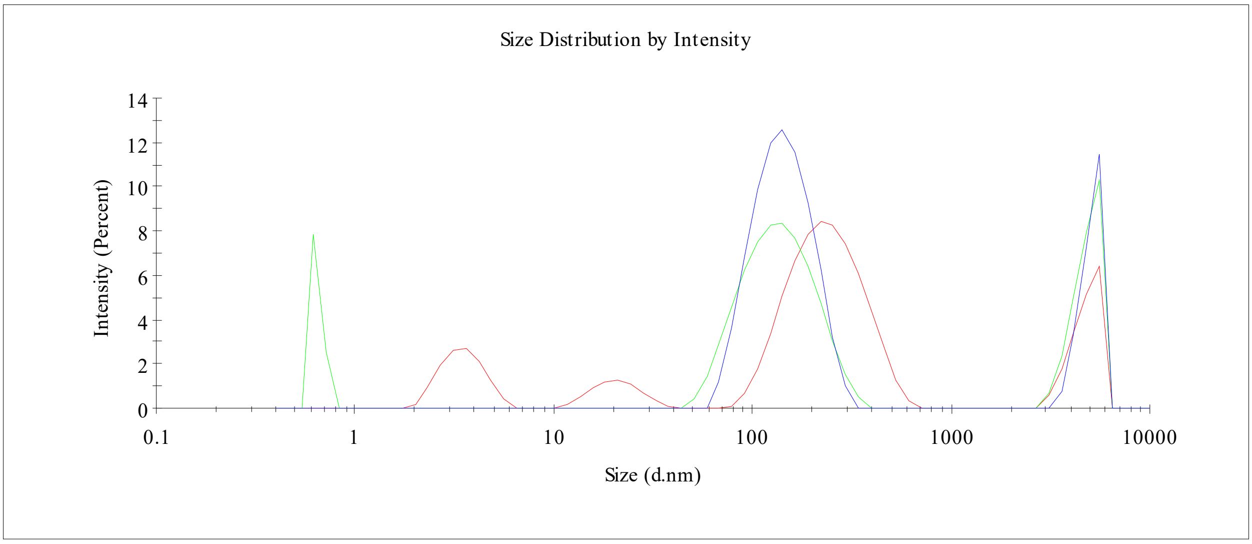

Figure S21. Size distribution of self-associates of macrocycle **3** in water (C_3=1 × 10^{-6} M).

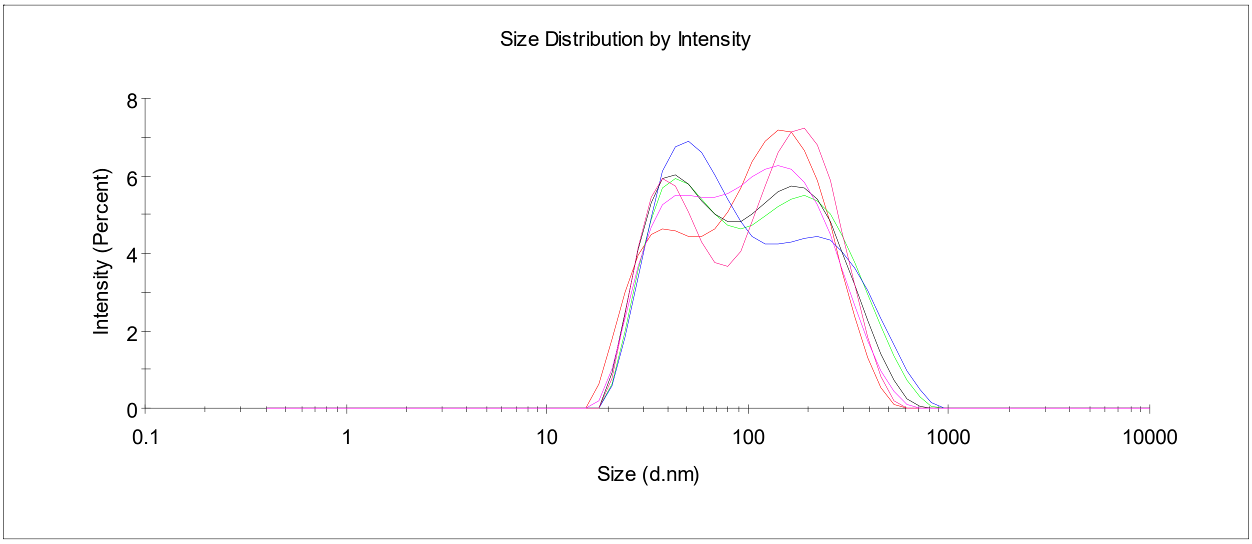

Figure S22. Size distribution of particles formed by **Fluo** (EtOH–H_2O, C_{Fluo}=1 × 10^{-4} M).

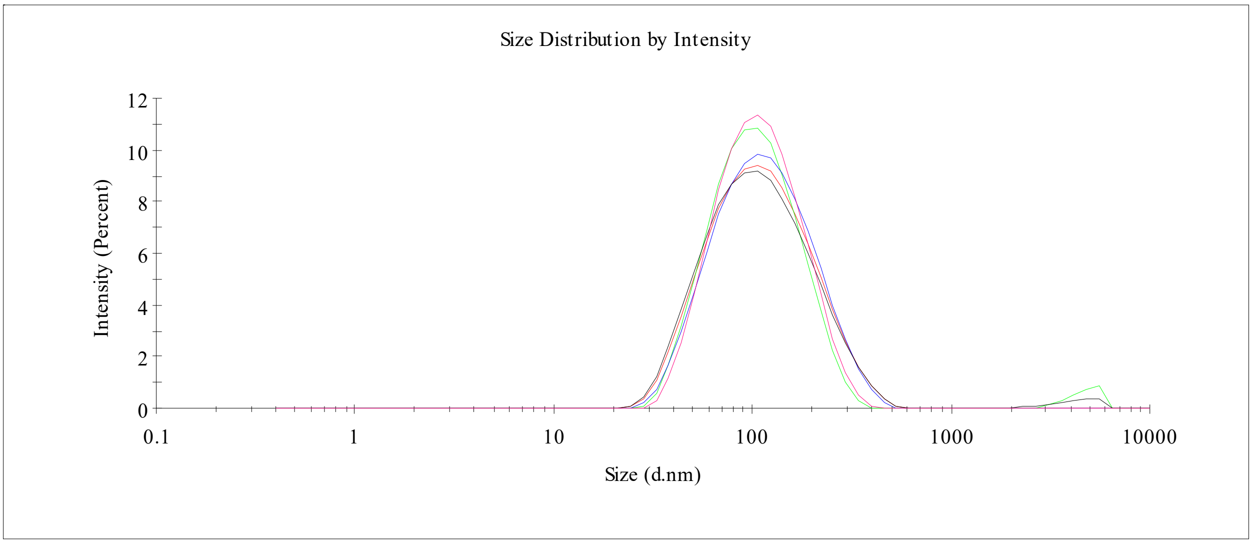

Figure S23. Size distribution of associates formed by macrocycle **2** and **Fluo** at 1:1 ratio (EtOH−H_2O, C=1 × 10^{-4} M).

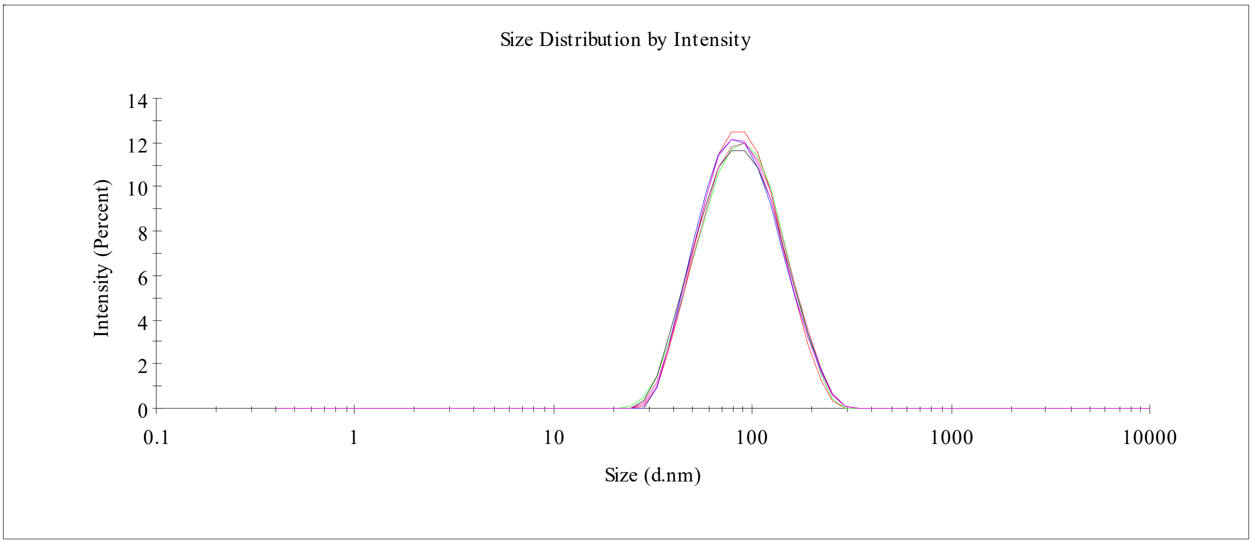

Figure S24. Size distribution of associates formed by macrocycle **3** and **Fluo** at 1:1 ratio ($EtOH–H_2O$, C=1 × 10^{-4} M).

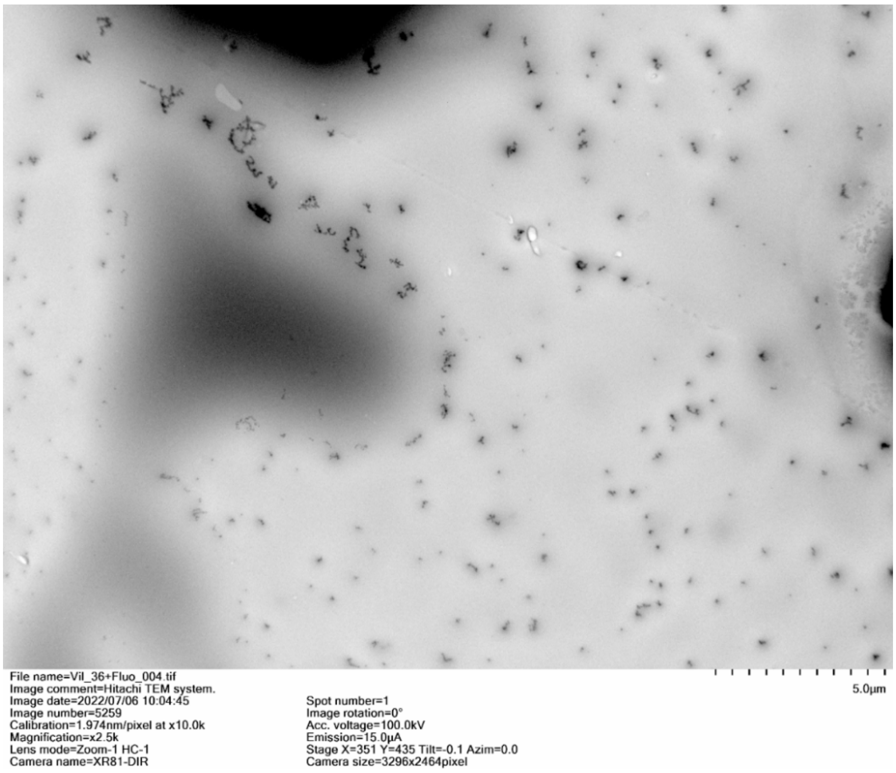

Figure S25. TEM image of aggregates formed by macrocycle **2** and **Fluo** at 1:1 ratio ($EtOH–H_2O$, C=1 × 10^{-4} M).

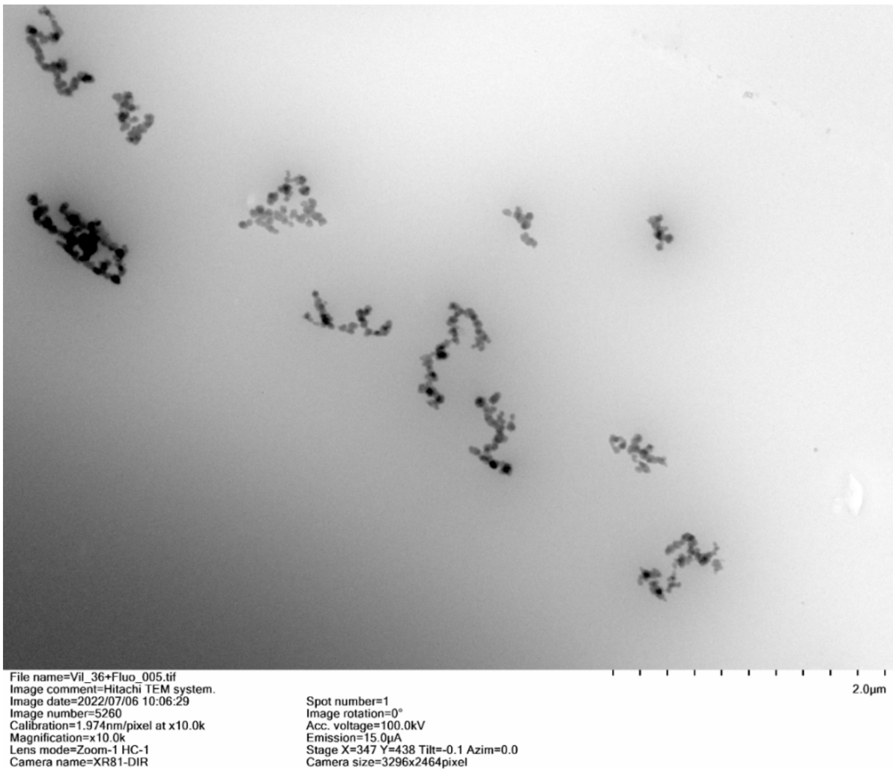

Figure S26. TEM image of aggregates formed by macrocycle **2** and **Fluo** at 1:1 ratio ($EtOH–H_2O$, C=1 × 10^{-4} M).

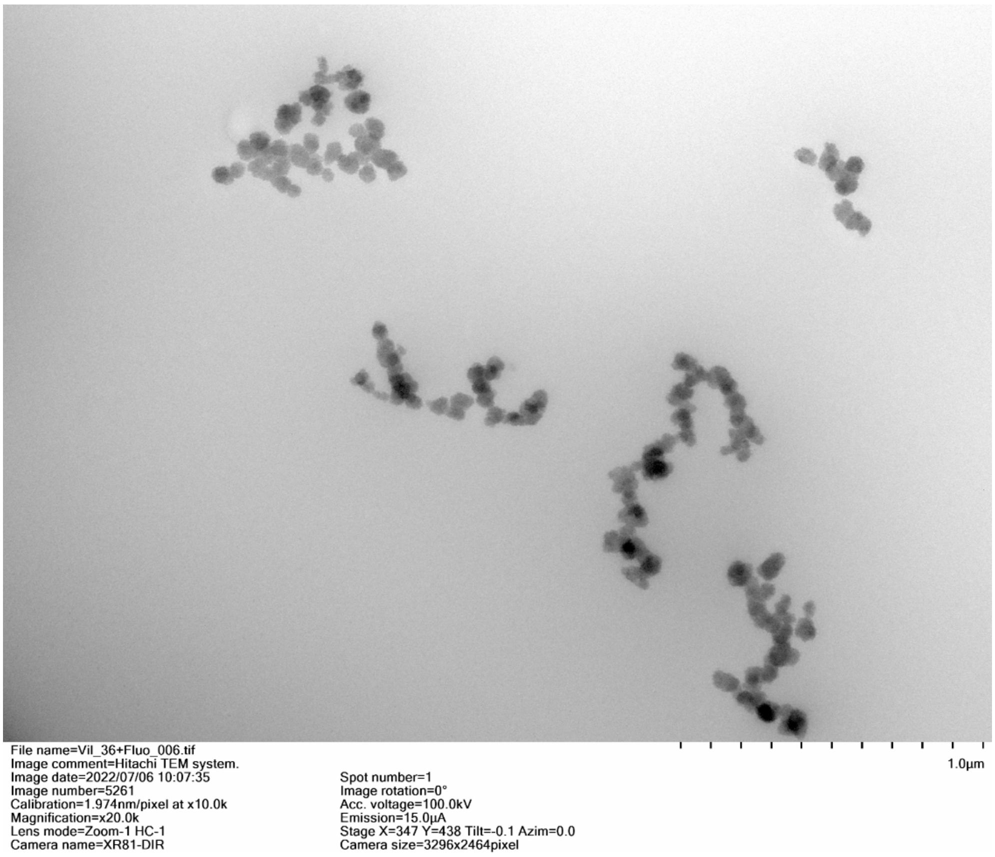

Figure S27. TEM image of aggregates formed by macrocycle **2** and **Fluo** at 1:1 ratio (EtOH–H_2O, C=1 × 10^{-4} M).

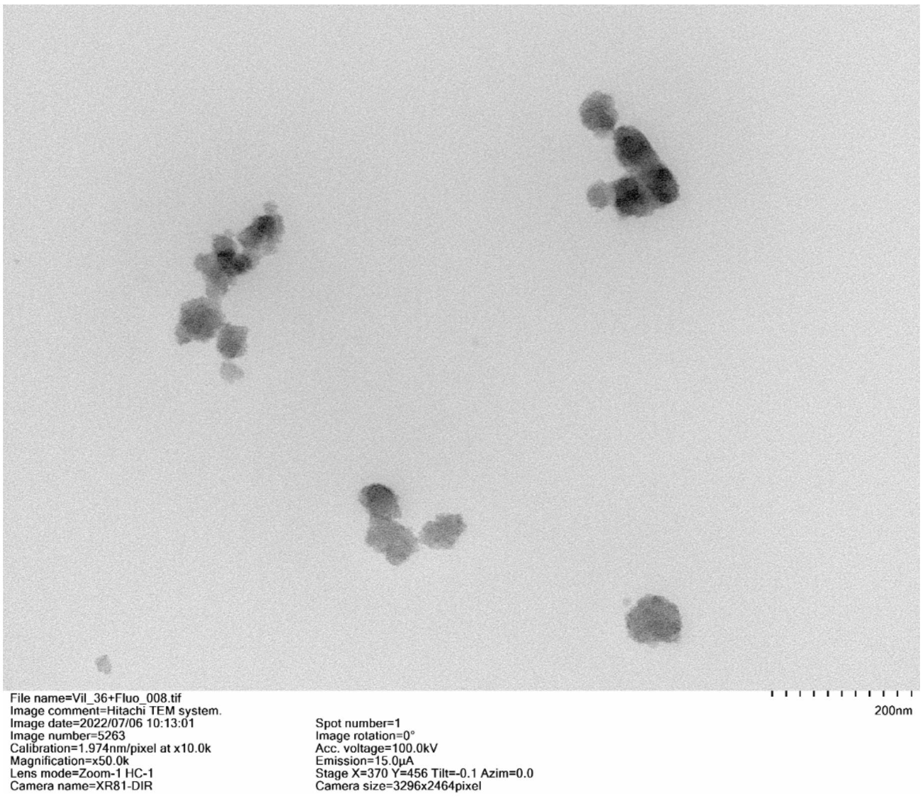

Figure S28. TEM image of aggregates formed by macrocycle **2** and **Fluo** at 1:1 ratio (EtOH–H_2O, C=1 × 10^{-4} M).

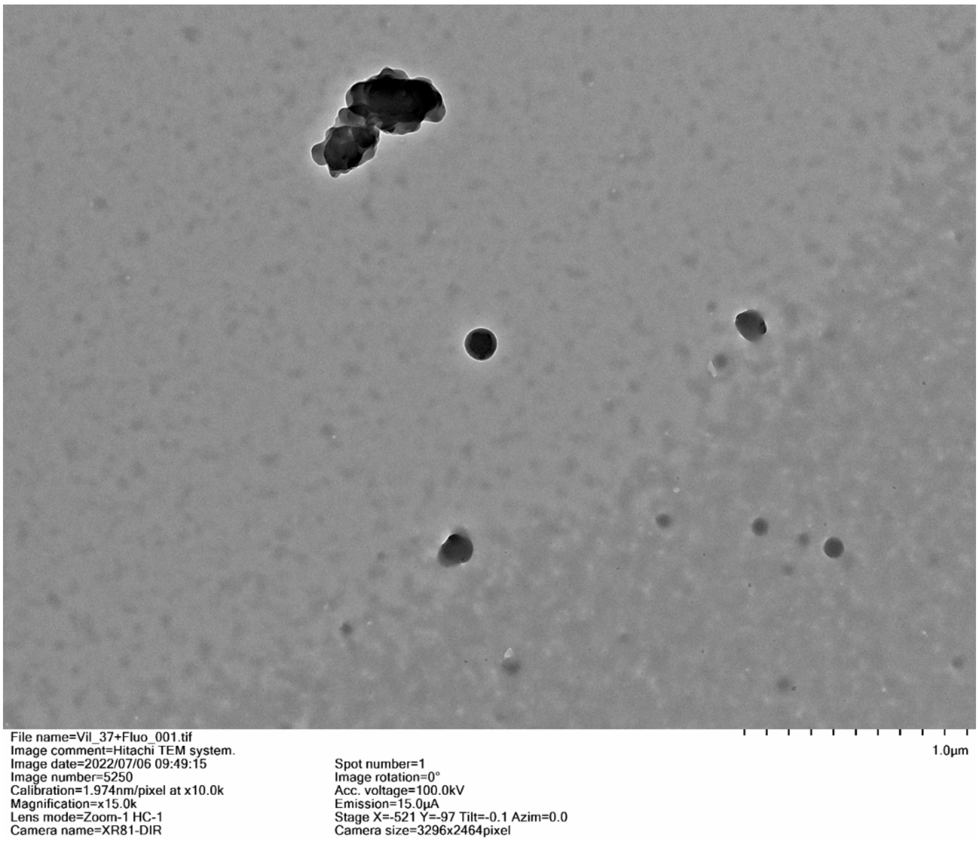

Figure S29. TEM image of aggregates formed by macrocycle **3** and **Fluo** at 1:1 ratio (EtOH–H_2O, C=1 × 10^{-4} M).

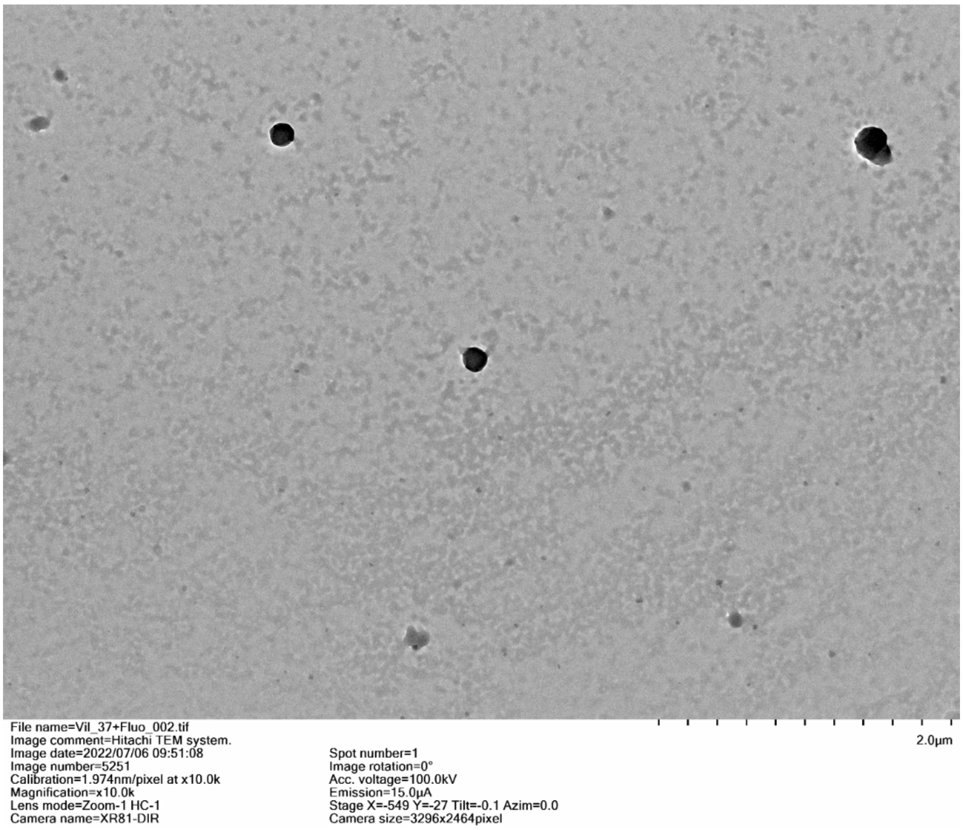

Figure S30. TEM image of aggregates formed by macrocycle **3** and **Fluo** at 1:1 ratio (EtOH–H_2O, C=1 × 10^{-4} M).

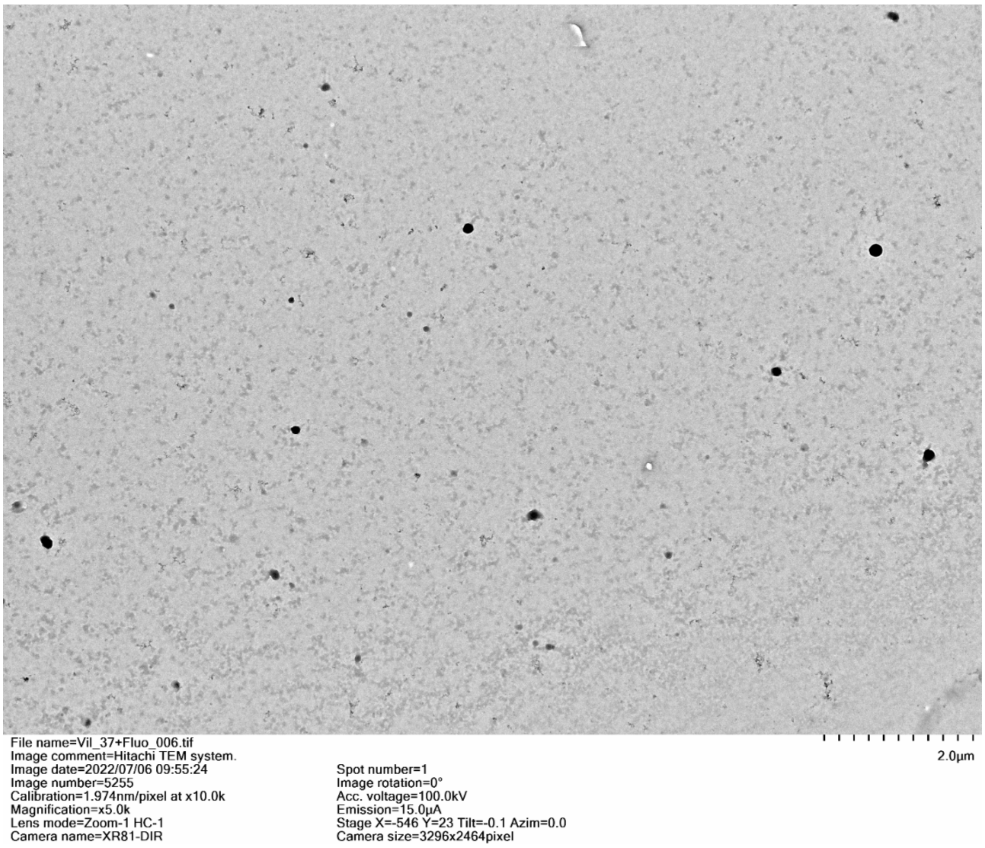

Figure S31. TEM image of aggregates formed by macrocycle **3** and **Fluo** at 1:1 ratio (EtOH–H_2O, C=1 × 10^{-4} M).

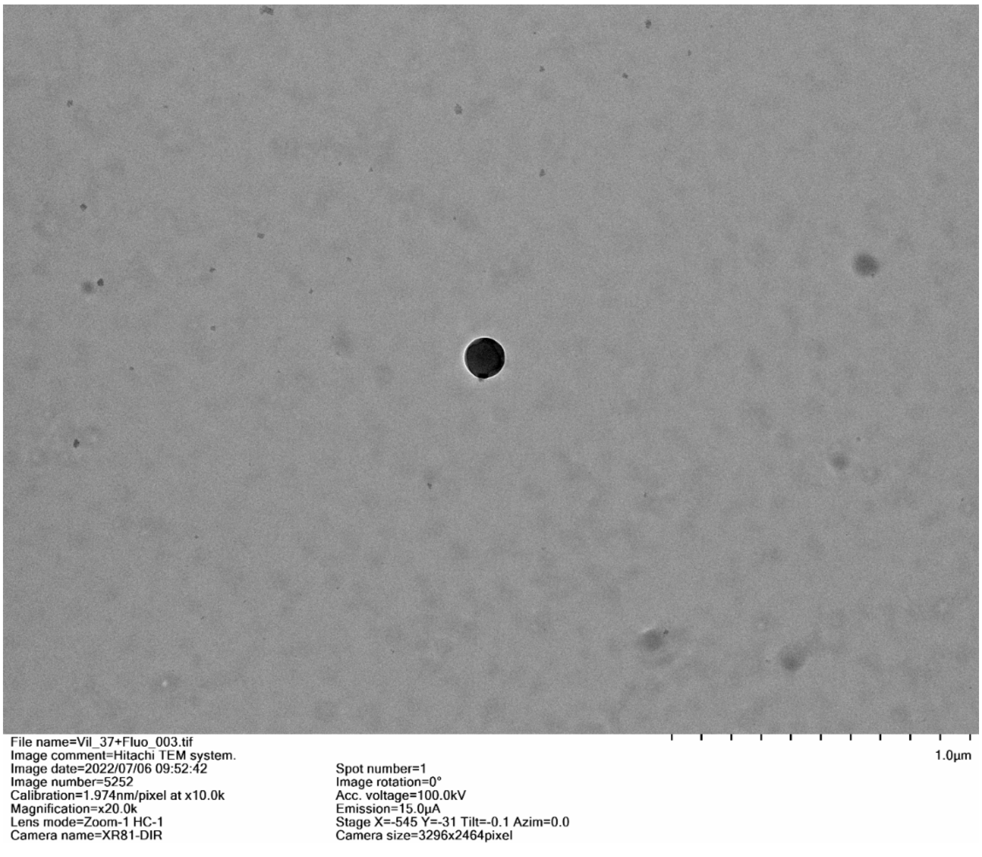

Figure S32. TEM image of aggregates formed by macrocycle **3** and **Fluo** at 1:1 ratio (EtOH–H_2O, C=1 × 10^{-4} M).

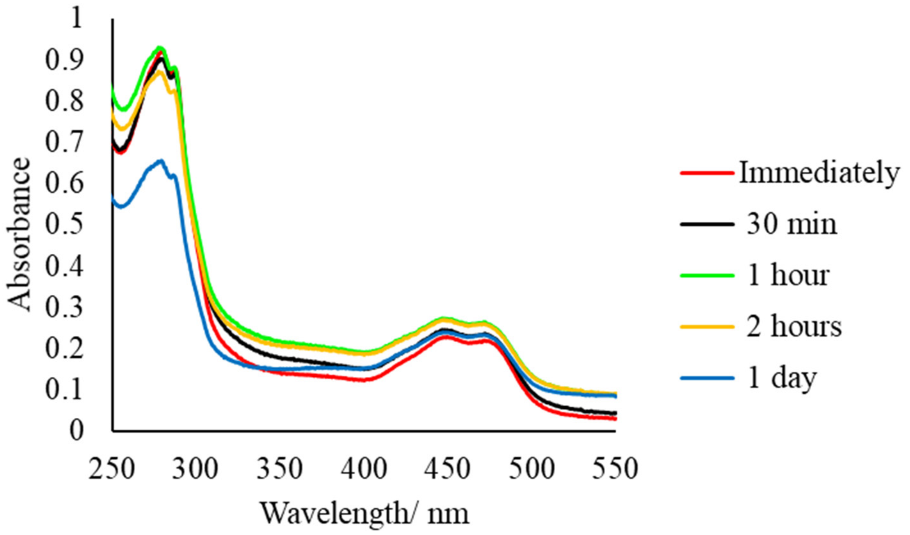

Figure S33. Electronic absorption spectra of **2/Fluo** in acetate buffer solution (pH=4.5) (C_2=1 × 10^{-5} M, C_{Fluo}=1 × 10^{-5} M).

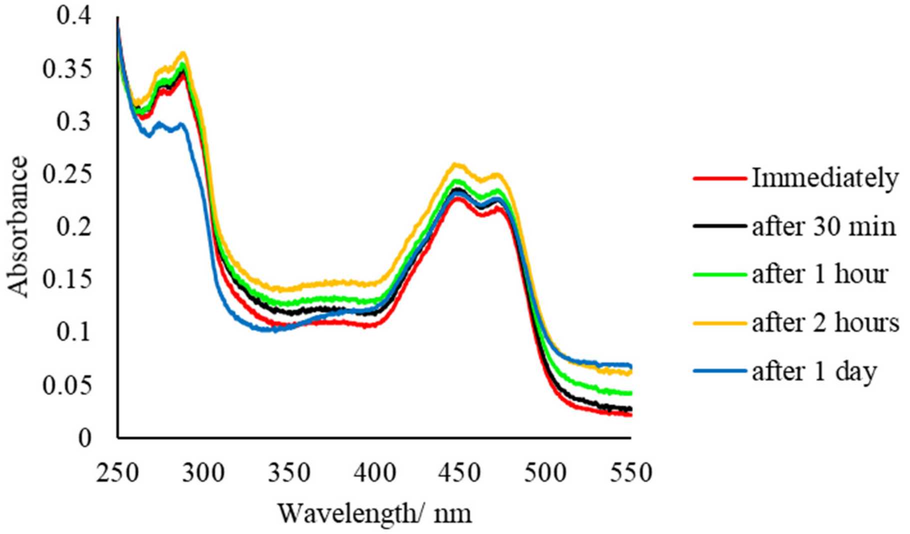

Figure S34. Electronic absorption spectra of **3/Fluo** in acetate buffer solution (pH=4.5) (C_3=1 × 10^{-5} M, C_{Fluo}=1 × 10^{-5} M).

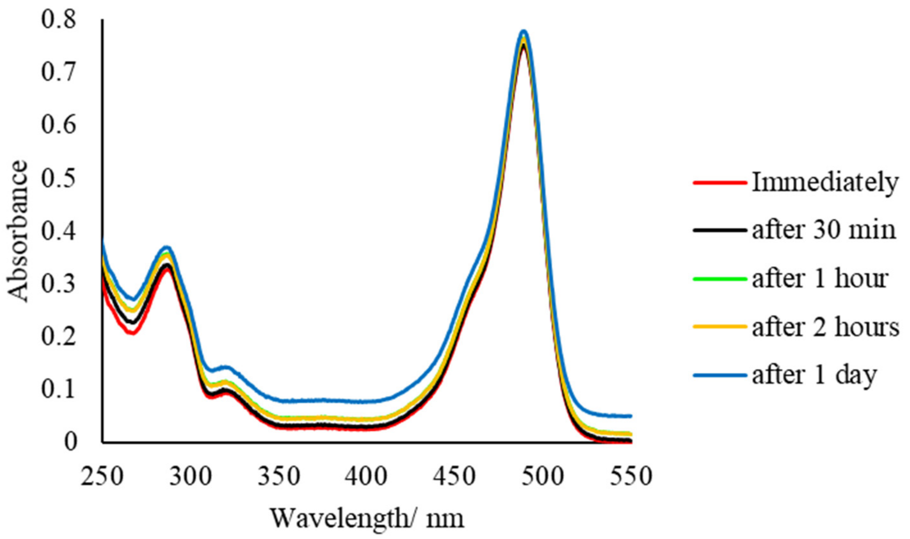

Figure S35. Electronic absorption spectra of **3/Fluo** in phosphate buffer solution (pH=7.4) (C_3=1 × 10^{-5} M, C_{Fluo}=1 × 10^{-5} M).

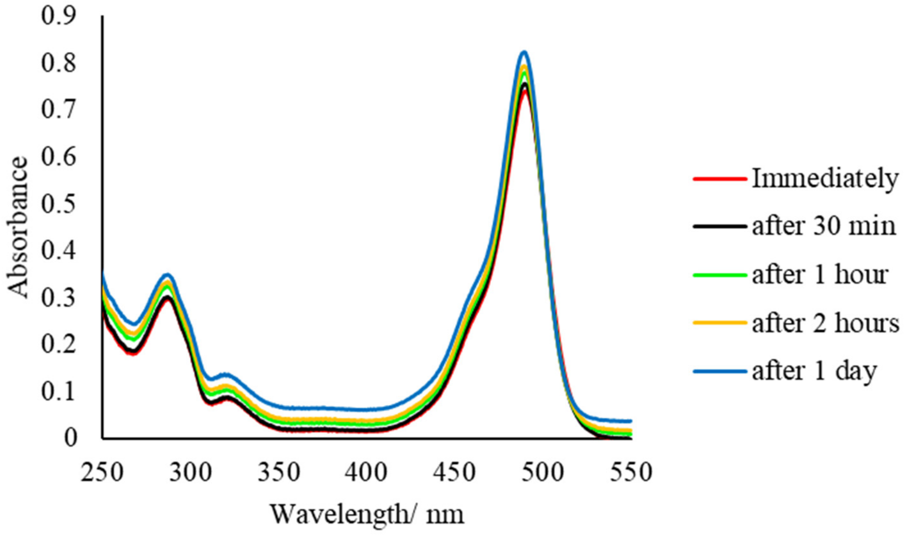

Figure S36. Electronic absorption spectra of **3/Fluo** in sodium tetraborate buffer (pH=9.2) (C_3=1 × 10^{-5} M, C_{Fluo}=1 × 10^{-5} M).

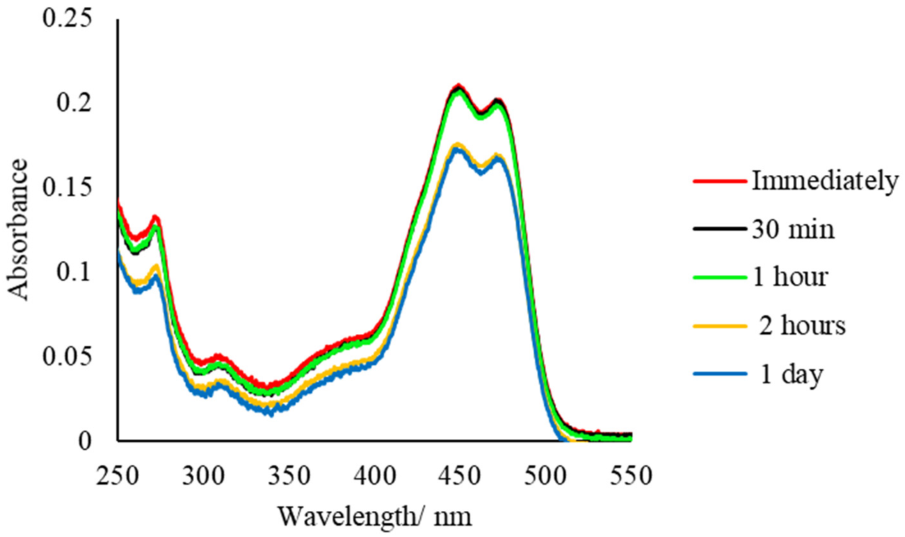

Figure S37. Electronic absorption spectra of **Fluo** in acetate buffer solution (pH=4.5) (C_{Fluo}=1 × 10^{-5} M).

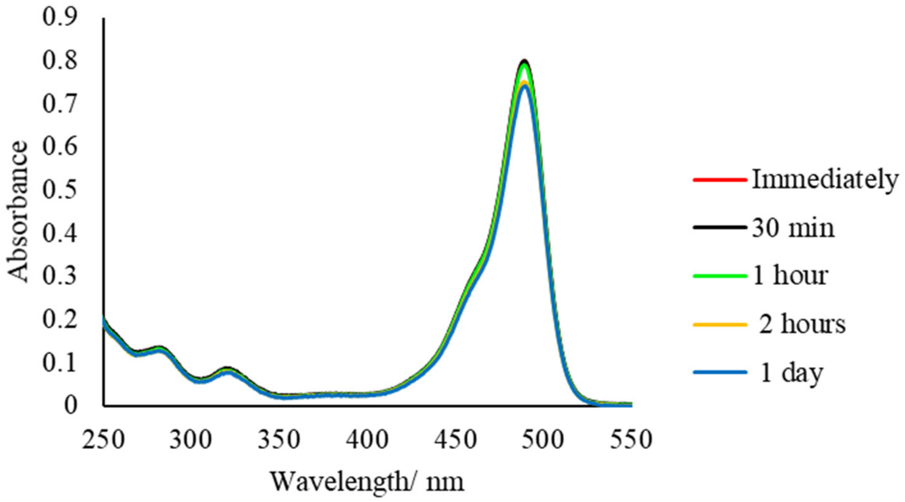

Figure S38. Electronic absorption spectra of **Fluo** in phosphate buffer solution (pH=7.4) (C_{Fluo}=1 × 10^{-5} M).

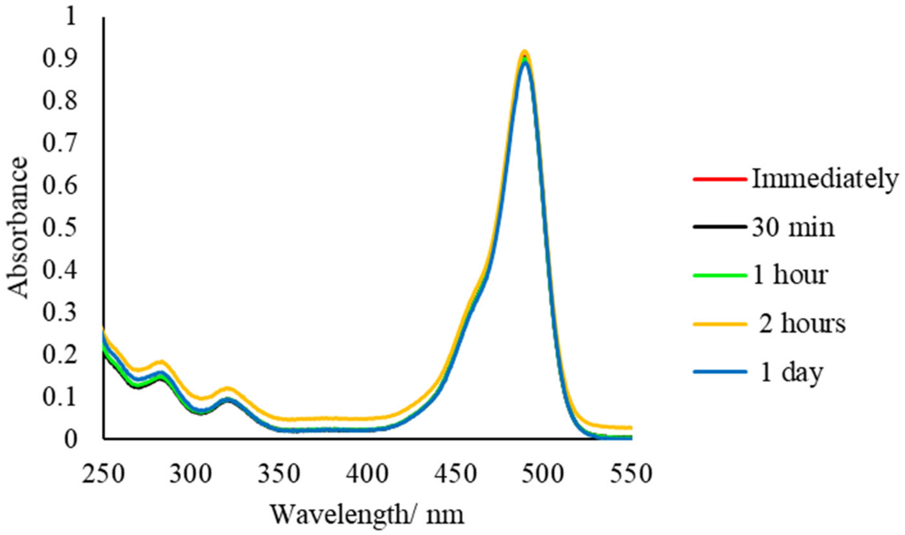

Figure S39. Electronic absorption spectra of **Fluo** in sodium tetraborate buffer (pH=9.2) (C_{Fluo}=1 × 10^{-5} M).

Table S3. The aggregates sizes (hydrodynamic particle diameters d, nm), intensity distribution of particles formed as a result of association **2** and **3** with **Fluo** in ethanol–water solution and diluted with different buffer solutions, polydispersity index (PDI) and zeta potential (ζ, mV).

Medium	**2/Fluo**			**3/Fluo**		
	PDI	d, nm	ζ, mV	PDI	d, nm	ζ, mV
pH=4.5	0.18±0.02	176±5	18±3	0.16±0.01	132±3	28±1
pH=7.4	0.75±0.10	1011±86	15±1	0.20±0.03	802±18	5±0
pH=9.2	0.41±0.03	248±46<br>38±5	13±2	0.19±0.01	634±36	32±1

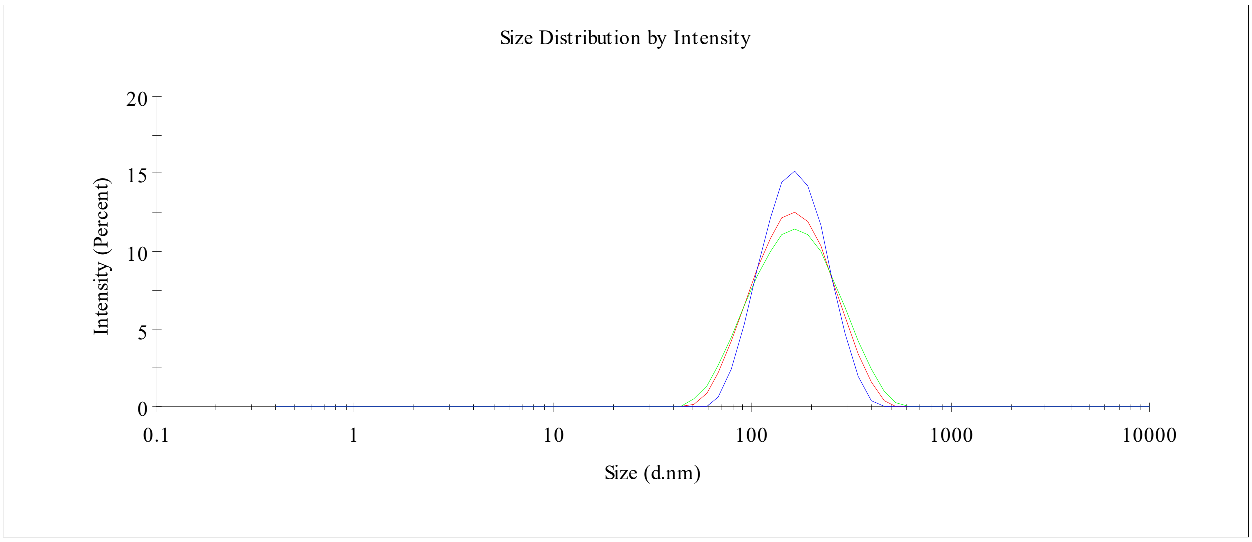

Figure S40. Size distribution of aggregates **2/Fluo** in acetate buffer solution (pH=4.5), C_2=1 × 10^{-5} M, C_{Fluo}=1 × 10^{-5} M.

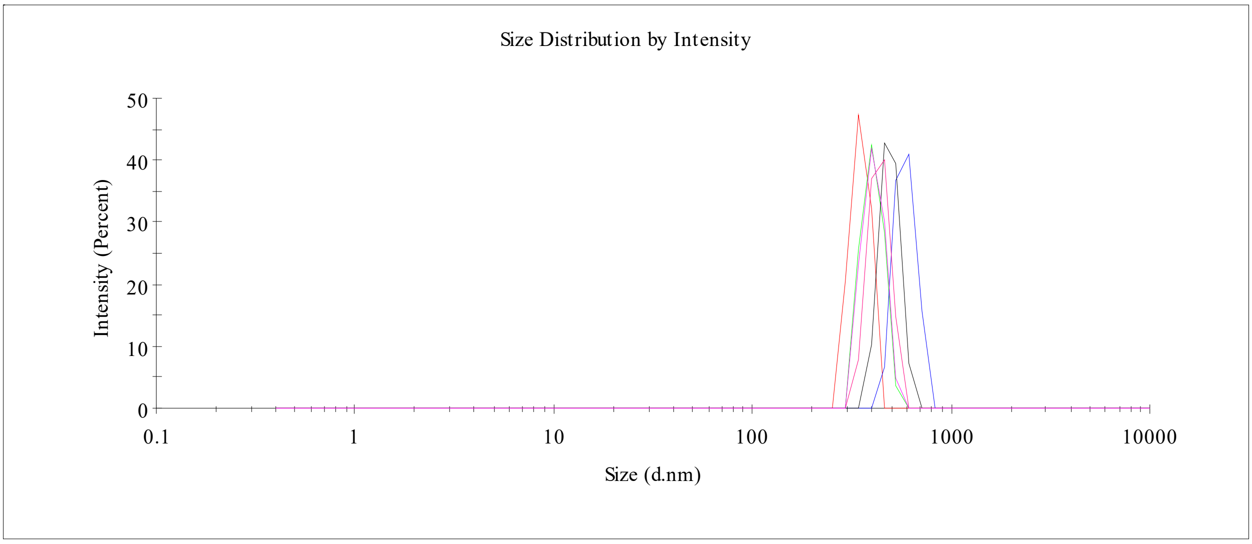

Figure S41. Size distribution of aggregates **2/Fluo** in phosphate buffer solution (pH=7.4), C_2=1 × 10^{-5} M, C_{Fluo}=1 × 10^{-5} M.

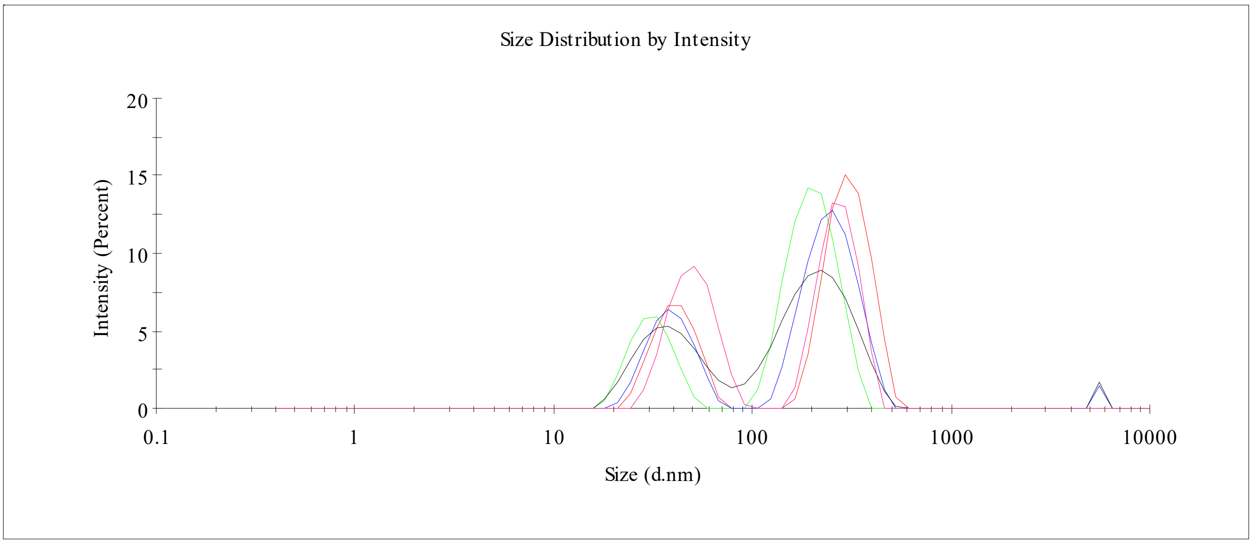

Figure S42. Size distribution of aggregates **2/Fluo** in sodium tetraborate buffer (pH=9.2), C_2=1 × 10^{-5} M, C_{Fluo}=1 × 10^{-5} M.

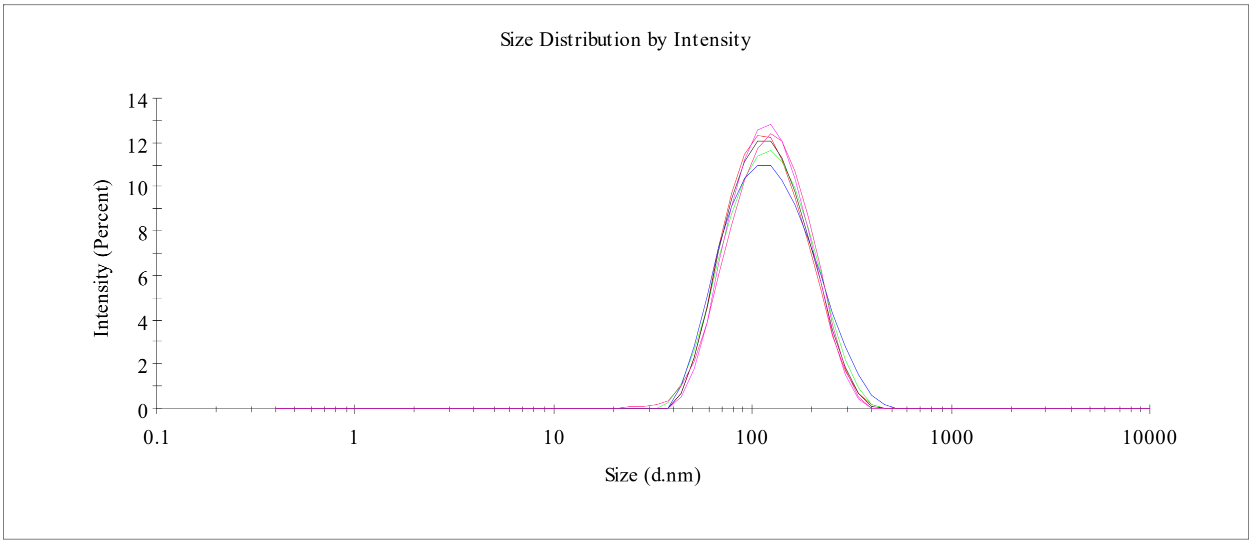

Figure S43. Size distribution of aggregates **3/Fluo** in acetate buffer solution (pH=4.5), C_3=1 × 10^{-5} M, C_{Fluo}=1 × 10^{-5} M.

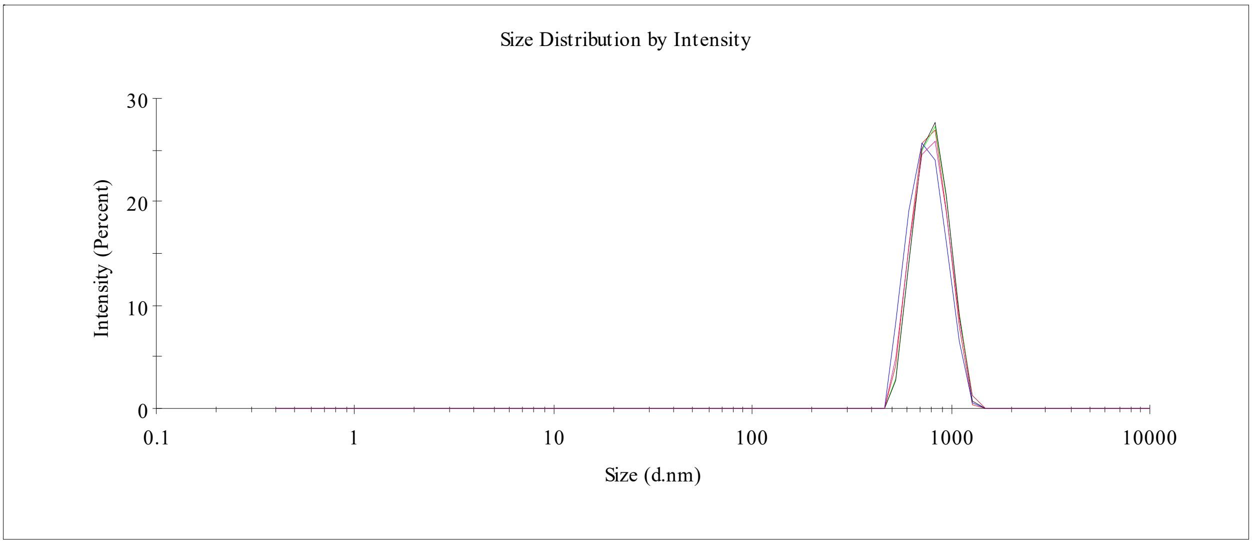

Figure S44. Size distribution of aggregates **3/Fluo** in phosphate buffer solution (pH=7.4), C_3=1×10^{-5} M, C_{Fluo}=1×10^{-5} M.

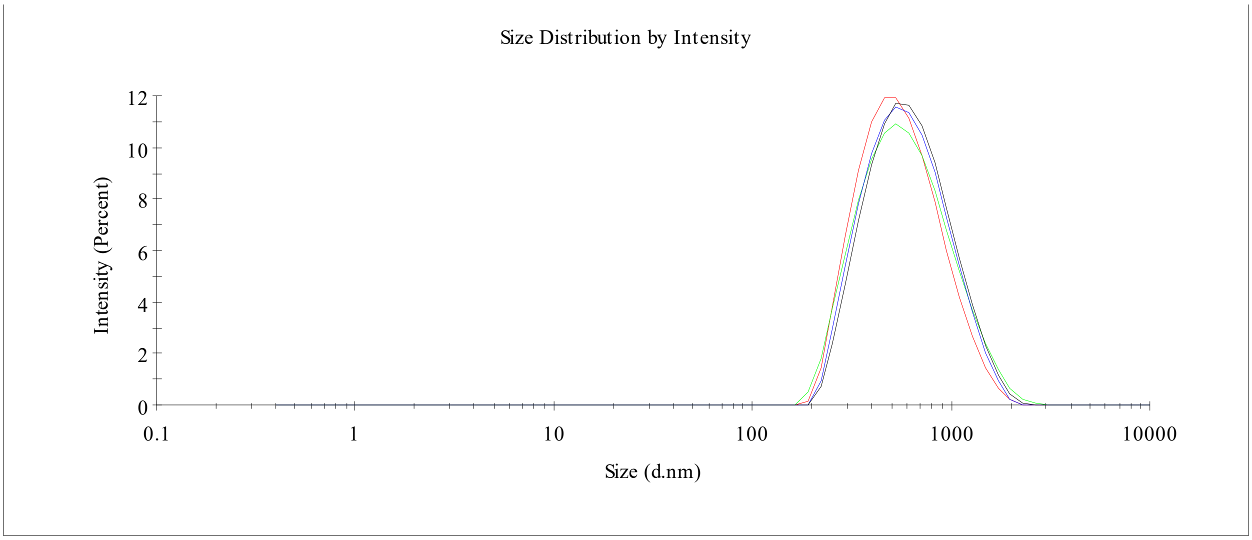

Figure S45. Size distribution of aggregates **3/Fluo** in sodium tetraborate buffer (pH=9.2), C_3=1×10^{-5} M, C_{Fluo}=1×10^{-5} M.

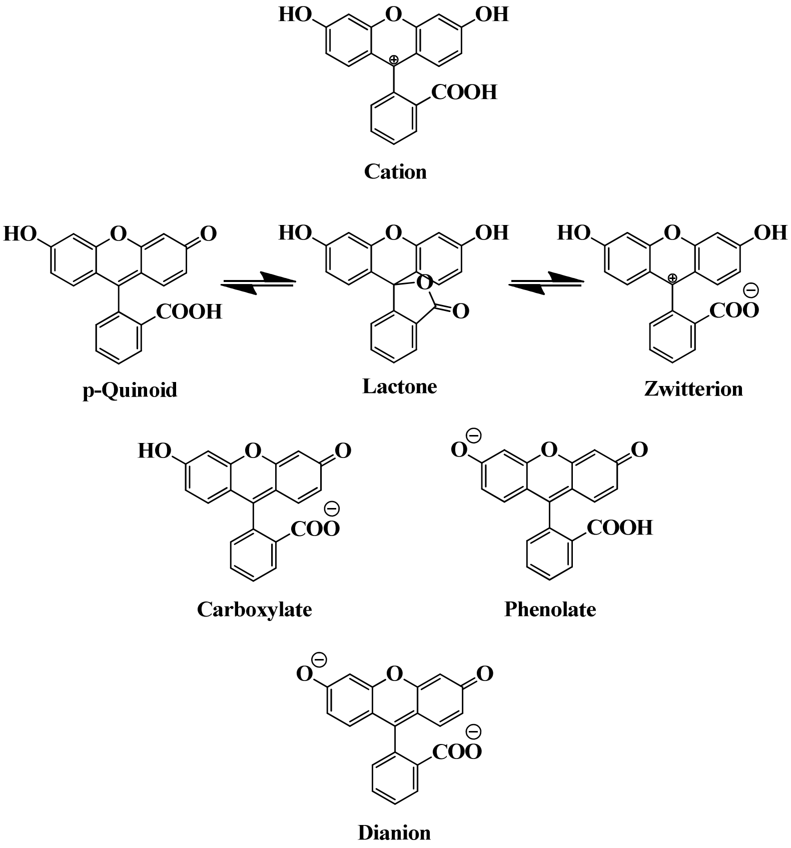

Figure S46. Chemical structures of fluorescein.
